# 3D bioprinting of tissues and organs for systemic diseases and localized injuries

**DOI:** 10.1016/j.mmr.2026.100006

**Published:** 2026-03-25

**Authors:** Wei Long Ng, Paulo Bartolo

**Affiliations:** Singapore Centre for 3D Printing, School of Mechanical and Aerospace Engineering, Nanyang Technological University, Singapore 639798, Singapore

**Keywords:** Biofabrication, Three-dimensional (3D) bioprinting, Bio-inks, Disease modelling, Tissue regeneration, Clinical translation, Multi-modal bioprinting, Machine learning (ML), Regulatory challenges

## Abstract

Three-dimensional (3D) bioprinting integrates engineering, materials science, and biology to fabricate living tissues with precise spatial control. By enabling the layer-by-layer deposition of cells and biomaterials, it overcomes many limitations of traditional scaffold-based tissue engineering and offers new opportunities for regenerative and personalized medicine. This review presents a comprehensive overview of recent advances in 3D bioprinting. It introduces a systematic, ASTM-aligned classification of key bioprinting modalities, extrusion, jetting, and vat photopolymerization, along with their respective material and biological design requirements. It also summarizes recent progress in bio-ink development and crosslinking strategies that improve print fidelity and functional tissue maturation. In addition, the review highlights applications in both systemic disease modelling and treatment (such as cardiovascular, endocrine/metabolic, and neurodegenerative disorders) and localized tissue repair (including skin, musculoskeletal, cartilage, and bone), emphasizing their relevance to civilian healthcare and military medicine. By combining technological innovation, biological insights, and regulatory considerations, this review outlines how advances in multi-modal bioprinting and intelligent process control can accelerate the translation of laboratory research into clinically viable, patient-specific therapies, driving the next generation of regenerative medicine.

## 1 Background

Tissue and organ failure resulting from ageing, trauma or disease remains one of the most pressing challenges in modern medicine [1]. Organ transplantation from living or deceased donors is currently the gold standard treatment for end-stage organ failure; however, the persistent shortage of suitable donor organs has reached a critical global level. According to the United Network for Organ Sharing, over 103,000 patients are currently on waiting lists for organ transplants in the United States alone, with more than 13 patients dying each day while awaiting a transplant [2]. Despite significant advances in surgical techniques and post-operative care, only a small fraction of patients receive life-saving organs due to donor scarcity. Even for those who undergo successful transplantation, lifelong immunosuppression therapy is typically required to prevent graft rejection, leading to severe side effects, increased infection risk, and substantial long-term healthcare costs [1].

In response to these limitations, regenerative medicine has emerged as a promising avenue for developing patient-specific replacement tissues and organs. The use of autologous cells or patient-derived stem cells offers a potential route to minimize immune rejection and reduce dependence on donor organs [3]. Early tissue engineering efforts focused on combining cells with biomaterial scaffolds to create functional constructs capable of restoring or replacing damaged tissues [4]. These scaffolds provided critical structural support, tunable mechanical properties, and controlled degradation rates and could be functionalized with bioactive cues to promote tissue development. However, conventional scaffold-based and cell-seeding methods have struggled to replicate the intricate spatial organization, vascular complexity, and cellular heterogeneity of native tissues. Challenges such as low initial cell density, non-uniform cell distribution, and limited ability to recreate hierarchical microarchitectures have limited the functionality, maturity, and scalability of engineered constructs [4].

To overcome these constraints, the field has increasingly turned to advanced biofabrication technologies, among which three-dimensional (3D) bioprinting has emerged as a transformative approach [5], [6]. 3D bioprinting enables the automated, layer-by-layer deposition of living cells, biomaterials, and signalling molecules with micrometre precision, allowing the fabrication of complex, cell-laden architectures that closely mimic the hierarchical organization and functional properties of native tissues [7]. This capacity for high spatial fidelity and multi-material integration provides unprecedented opportunities to engineer physiologically relevant tissues that integrate both structural and functional complexity. Nevertheless, key challenges persist, including the need to reproduce organ-specific microarchitecture, establish perfusable vascular networks, and maintain long-term tissue functionality both *in vitro* and *in vivo* [8]. Furthermore, ensuring reproducibility, scalability, and compliance with regulatory and manufacturing standards remains a significant obstacle to clinical translation [9].

Given these scientific and translational challenges, this review critically examines the current landscape of 3D bioprinting for tissue and organ fabrication. We summarize recent advances in bioprinting modalities, bio-ink development, and state-of-the-art applications in the fabrication of various tissues and organs targeting both systemic diseases and localized injuries. Additionally, we analyze the remaining limitations that hinder large-scale, clinically relevant implementation and highlight emerging directions, including multi-material bioprinting, machine learning (ML)-assisted optimization, and regulatory standardization that will shape the future trajectory of this rapidly evolving field toward clinical realization.

## 2 Bioprinting techniques

3D bioprinting has become a transformative tool in tissue engineering, enabling the deposition of living cells and biomaterials with precise spatial control. By replicating both the anatomical complexity and functional microarchitecture of native tissues, 3D bioprinting provides capabilities far beyond those of traditional scaffold-based approaches [9], [10]. Current bioprinting techniques are broadly categorised into three main modalities, extrusion-based [11], [12], [13], jetting-based [14], [15], [16], [17], and vat photopolymerization-based systems [18], [19], [20], [21], each offering unique advantages and facing specific limitations. A comprehensive understanding of these techniques is essential for selecting appropriate fabrication methods, optimizing process parameters, and guiding the development of hybrid systems for clinically translatable tissue constructs.

### 2.1 Extrusion-based bioprinting

**Fig. 1:**
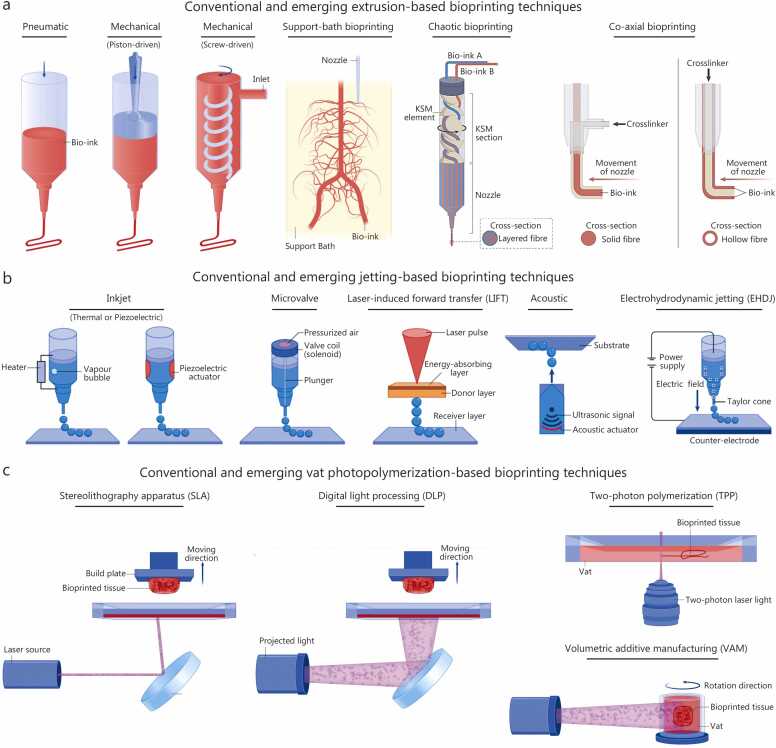
Overview of major bioprinting techniques. **a** Extrusion-based bioprinting utilizing pneumatic or mechanical dispensing systems, with advanced variants such as support-bath, chaotic, and coaxial bioprinting. Reproduced with permission [11]. **b** Jetting-based modalities, including inkjet (thermal or piezoelectric), microvalve, laser-induced forward transfer, acoustic, and electrohydrodynamic jetting. Reproduced with permission [15]. **c** Vat photopolymerization-based approaches, encompassing stereolithography apparatus, digital light processing, two-photon polymerization, and volumetric additive manufacturing (VAM). Reproduced with permission [21].

Extrusion-based bioprinting is the most widely adopted approach due to its versatility, ability to process bio-inks across a broad viscosity range, and suitability for fabricating large, mechanically robust, and anatomically relevant constructs (Fig. 1**a**) [11]. The technique relies on either pneumatic or mechanical dispensing systems to extrude bio-inks continuously. Pneumatic systems use pressurized air but are prone to pressure fluctuations, whereas mechanical systems employing piston- or screw-driven configurations offer improved flow control at the expense of increased shear stress on encapsulated cells [22]. Balancing shear stress, nozzle geometry, and crosslinking kinetics is therefore critical for achieving high printing fidelity without compromising cell viability [23], [24].

Despite its versatility, conventional extrusion-based bioprinting faces several notable limitations. Low-viscosity bio-inks often exhibit poor shape fidelity, making it difficult to maintain fine structural features, and achieving microscale architectural precision (< 100 µm) remains challenging [11]. Recreating biomimetic tissue microenvironments and integrating perfusable vascular networks is particularly difficult for thick or multi-layered constructs [25]. Additionally, shear stress during extrusion can reduce cell viability by 10%–40%, depending on nozzle diameter, flow rate and bio-ink viscosity, which limits the use of sensitive or primary cell types [23], [24]. These limitations highlight the need for complementary strategies, such as support-bath, chaotic, or coaxial bioprinting, to improve both structural fidelity and biological functionality.

Several variants have emerged to address these limitations. Support-bath bioprinting allows high-fidelity deposition of low-viscosity bio-inks by extruding them within a yield-stress medium that provides temporary mechanical support during printing. Such support baths can be broadly classified into particle-based systems, typically composed of hydrogel microparticles (e.g., gelatin, alginate, agarose, Carbopol) compacted into a jammed state, and continuous-phase systems formed from shear-thinning, self-healing hydrogels (e.g., cellulose-based, hyaluronic acid, Pluronic F-127, or xanthum gum) [26]. While hydrogels and microparticles are distinct entities, hydrogels can be engineered into microparticles that collectively exhibit reversible solid-liquid transitions, enabling smooth nozzle movement and immediate self-recovery of the medium [26]. The support bath thus behaves as a viscoelastic Bingham plastic, flowing under shear but quickly self-healing afterwards to preserve the printed structure’s integrity [26]. Critical factors influencing print fidelity include the bath’s yield stress, rheological recovery, compatibility with diverse crosslinking mechanisms, and controlled liquefaction for non-destructive construct retrieval [27]. The process is further governed by microparticle size and uniformity, as well as interfacial and gravitational stability, osmotic pressure, nozzle geometry, printing speed, flow rate, and post-processing conditions [28]. Notably, particle size plays a critical role in determining resolution: smaller, monodisperse microparticles (about 25 µm) produced by complex coacervation yield filament resolutions as fine as approximately 20 µm [29], whereas coarser or blended microparticles (about 60 µm) result in lower resolution [30]. Support baths also facilitate high-density cell printing (approximately 3.0×10^8^ million cells/ml) and anisotropic tissue fabrication through shear-induced cell alignment [29]. Crosslinking within the bath may occur through pH modulation, ionic interactions, enzymatic reactions, or photopolymerization, provided these mechanisms do not interfere with the support matrix [27]. Beyond serving as a printing medium, hydrogel microparticles can also function as dual-purpose materials, acting both as bio-inks for direct extrusion and as self-healing matrices for support-bath bioprinting [31], [32]. The resulting bioprinted constructs can be retrieved using non-destructive release strategies, methods for removing the 3D bioprinted constructs from the support bath, ensuring the integrity and fidelity of the printed structures, depending on bath composition, such as thermal transitions (gelatin) [33], ion exchange (Carbopol) [34], or enzymatic/chemical triggers (alginate/xanthan gum) [35]. Future developments are expected to focus on stimuli-responsive and reusable baths, optimized retrieval methods, and advanced slicing algorithms to further enhance omnidirectional, freeform bioprinting.

Chaotic bioprinting leverages deterministic chaotic advection within a Kenics static mixer (KSM) printhead to generate highly aligned, multi-material microstructures [36]. Helicoidal elements inside the printhead repeatedly split and reorient bio-ink streams, doubling the number of layers with each KSM to create intricate internal architectures without subjecting cells to excessive shear stress [37]. Successful bioprinting depends on carefully tuning bio-ink rheology and interfacial properties to preserve well-defined interfaces, as well as optimizing nozzle diameter, ionic strength, and crosslinking timing [37]. Current approaches commonly extrude alginate-based bio-inks into calcium chloride (CaCl_2_) baths [38], [39], or employ *in situ* photo-crosslinking for gelatin methacryloyl (GelMA)-based bio-inks [39], [40]. Future improvements could focus on broadening material compatibility, increasing the number of KSM elements, and integrating multiple inlet ports to further enhance the structural and compositional complexity of bioprinted constructs [40].

Co-axial bioprinting employs concentric nozzles to extrude core-shell configurations, enabling the fabrication of perfusable tubular structures such as vascular channels [41]. Variants including tri- and quad-axial systems require precise control of flow rates, crosslinking kinetics, and shell mechanical stability to maintain lumen patency [42], [43]. Sacrificial cores such as gelatin, polyvinyl alcohol (PVA), and Pluronic F127 [44], [45] or ionically crosslinkable alginate shells are commonly used [46], [47], with parameters such as nozzle alignment and flow rate ratios critical for reproducible printing outcomes.

In summary, extrusion-based bioprinting offers unmatched versatility for large-scale, multi-material, and cell-laden constructs, yet its major limitations include resolution constraints, shear-induced cell damage, and difficulty in replicating native tissue microarchitecture and vasculature. Variants such as support-bath, chaotic, and co-axial bioprinting provide promising solutions, each addressing specific shortcomings while introducing new complexities in process control, scalability, and material compatibility. Future developments will likely depend on integrating adaptive crosslinking strategies, real-time process monitoring, and advanced bio-ink formulations to reconcile printing fidelity with biological functionality, moving closer to the scalable fabrication of clinically relevant tissues and organ-level constructs.

### 2.2 Jetting-based bioprinting

Jetting-based bioprinting operates on a fundamentally different principle, employing non-contact, drop-on-demand (DOD) deposition of picolitre- to nanolitre-sized droplets with exceptional spatial precision (Fig. 1**b**) [15]. This enables fine control over cell placement and cell-cell interactions, making it particularly suited for applications requiring high-resolution patterning rather than bulk volume fabrication. Common jetting modalities include thermal and piezoelectric inkjet bioprinting [48], microvalve-based systems [49], laser-induced forward transfer (LIFT) [50], microfluidics-based [51], and acoustic-based bioprinting [52]. Successful droplet-based bioprinting depends on the interplay between surface tension, viscosity, and inertia of the bio-ink, which can be described by the dimensionless Ohnesorge number (*Oh*) [53]. The *Oh* defines the “printability window” for stable droplet formation: low *Oh* (<0.1) can lead to satellite droplet formation, while high *Oh* (>1) suppresses droplet ejection due to excessive viscous damping. Jetting-based bioprinting typically achieves printing resolutions up to about 100 µm, depending on nozzle size and actuation frequency, and is generally suitable for fabricating tissue constructs up to a few millimetres thick, limited by coalescence of low-viscosity ink droplets. Ensuring high cell viability requires careful optimization of droplet velocity, shear stress, and ink formulation [54], [55], [56], [57].

While jetting-based systems excel in precision, their inability to produce thick, mechanically robust constructs limits their standalone applicability. Among emerging variants, electrohydrodynamic jetting (EHDJ) bioprinting, also known as bio-electrospraying, offers distinct advantages in achieving high resolution. Pioneering study demonstrated the feasibility of directly electrospraying living cells and biomaterials under high electric fields, establishing the foundation for EHDJ bioprinting [58]. The early studies elucidated how cells could survive electrostatic jetting and how jet parameters govern cell encapsulation, distribution, and post-print viability. These insights are critical for modern EHDJ-based bioprinting platforms [58]. EHDJ employs a high-voltage electric field (0.5–30 kV) between the nozzle and substrate to overcome surface tension, deforming the bio-ink meniscus into a Taylor cone from which droplets are ejected. By relying on electrostatic forces rather than mechanical pressure, EHDJ bioprinting utilized small nozzles (60 µm) to generate high-resolution cell-laden filaments (30–50 µm), while reducing lateral spread and mechanical stress on cells [59], [60]. Key parameters influencing EHDJ bioprinting include bio-ink properties (surface tension, viscosity, electrical conductivity, and evaporation rate), hardware settings (nozzle dimensions, applied voltage, and flow rate), and substrate characteristics (wettability and electrical properties) [61], [62]. Droplet formation occurs in modes governed by electric field strength (E) and flow rate (Q) [63]: 1) dripping mode (low E and Q), 2) pulsating mode (moderate E and Q), 3) cone-jet mode (high E; ideal for precision), and 4) spray mode (excessively high E; unstable). Additional parameters such as the direction of electric charge flow [64], field polarity [65], substrate surface chemistry and conductivity [66], and nozzle material properties [67] also influence printing outcomes. While EHDJ bioprinting offers superior resolution, challenges remain in broadening the range of printable bio-inks and scaling to larger constructs [68].

Recent advances have extended jetting-based principles to the single-cell scale [69], [70], where microfluidics and LIFT mechanisms enable deterministic placement of individual cells with micrometre precision. A high-definition single-cell printer integrating microfluidic droplet generation with fluorescence-activated sorting and dielectrophoretic control achieves >99.5% single-cell ejection probability toward a substrate with about 10 µm spatial accuracy [71]. This approach enabled rapid (about 100 Hz) and highly viable (>94.0%) single-cell printing, allowing programmed construction of cellular arrays, tissue-like patterns, and uniform multicellular spheroids with controlled composition and morphology. Complementarily, another study demonstrated a femtosecond laser-induced single-cell bioprinting technique that relies on localized optical breakdown within a hydrogel to generate cavitation-driven microjets that transfer selected cells onto a receiver substrate [72]. This technique enables precise, non-contact single-cell transfer with minimal thermal or mechanical damage. Together, these innovations bridge microfluidic control and jetting physics to achieve true single-cell resolution, advancing applications in organoid assembly, cellular heterogeneity studies, and tissue microarchitecture engineering. Jetting-based bioprinting provides unmatched resolution for patterned cell deposition but is constrained by throughput and mechanical limitations, motivating interest in techniques capable of combining precision with volumetric complexity.

### 2.3 Vat photopolymerization-based bioprinting

Vat photopolymerization-based bioprinting offers some of the highest spatial resolutions (about 30 µm) among current bioprinting modalities, enabling the fabrication of intricate surface topographies, hierarchical architectures, and finely organized cell arrangements [73]. In this approach, photo-crosslinkable bio-inks (bio-resins) within a vat are selectively irradiated to induce localized photopolymerization. Common variants include stereolithography apparatus, digital light processing (DLP), and two-photon polymerization (Fig. 1**c**) [21].

The bio-inks used in these systems generally comprise photo-active monomers, photo-initiators (PIs) such as Irgacure 2959 or lithium phenyl-2,4,6-trimethylbenzoylphosphinate (LAP) [18], and photo-absorbers (PAs) like ponceau 4 R or tartrazine to modulate light penetration and crosslinking depth [74]. To maintain uniform cell distribution during printing, buoyancy-matching additives such as Percoll [75] or high molecular weight photopolymerizable precursors like sodium hyaluronate [76] are often incorporated. Without these additives, the heavier cells tend to sediment over time under gravitational forces, resulting in non-uniform cellular distribution within the printed construct [18]. By balancing the density difference between cells and the surrounding matrix, buoyancy-matching components help preserve a homogeneous suspension and ensure consistent cell placement throughout the bioprinting process [18].

Cell viability during vat photopolymerization depends strongly on the light source parameters (wavelength, intensity, and exposure duration) and the PIs type and concentration [77]. Excessive exposure or high photon flux can elevate free radical generation and localized heating, inducing oxidative stress, membrane disruption, or deoxyribonucleic acid (DNA) damage [78]. Cytotoxicity may also arise from unreacted PIs residues or reactive oxygen species (ROS) generated during crosslinking [79]. To mitigate these effects, biocompatible PIs such as Irgacure 2959 or LAP are typically used at low concentrations with near-visible light sources (365–405 nm) [79]. Optimization of exposure time, light intensity, and resin formulation can further minimize phototoxicity and preserve cellular function and proliferation.

Recent innovations have significantly advanced vat photopolymerization. Continuous liquid interface production (CLIP) utilizes an oxygen-permeable window to create a non-polymerizing “dead zone”, enabling continuous, layer-free printing at speeds exceeding 1000 mm/h [80], [81]. The thickness of the “dead zone” and cured layers is precisely tuned by photon flux, PIs absorptivity, and bio-resin absorption coefficients. Although CLIP has been extensively applied in biomedical engineering, its use for bioprinting live cells is still emerging. To date, only one study has demonstrated successful CLIP bioprinting of cell-laden constructs, in which the bioprinted cells proliferated over two weeks in culture with high viability [82]. Molecular analyses revealed only a mild, statistically insignificant increase in ROS and DNA damage compared to untreated controls, indicating that CLIP can support live-cell printing with minimal cytotoxicity [82].

Injection-CLIP improves upon this by actively injecting resin into the print zone, supporting higher-viscosity inks (3–5×higher than CLIP) and multi-material printing [83]. Volumetric additive manufacturing reconstructs entire 3D volumes in seconds by projecting sequences of two-dimensional (2D) images through a rotating vat, achieving feature sizes of approximately 30 µm and high cell viability (≥85%) [84]. Despite these advances, challenges such as limited bio-resin availability, optical scattering, and high costs persist. Strategies to address these issues include refractive index-matching agents (e.g., iodixanol [85], [86], iohexol [87]) and novel methods such as filamented light (FLight) bioprinting, which uses optical modulation instability to create aligned microfilament networks with ultra-high aspect ratios [88], [89]. Vat photopolymerization-based bioprinting offers unparalleled resolution and structural complexity, making it a powerful complement to extrusion-based and jetting-based bioprinting techniques in the pursuit of functional, clinically relevant tissue constructs.

### 2.4 Comparative perspective on bioprinting techniques

Extrusion-based bioprinting remains the most widely adopted modality due to its versatility, capacity to deposit high-viscosity bio-inks, and ability to fabricate robust, clinically relevant-sized constructs [11]. In this context, a clinically relevant construct refers to a size sufficient to restore or bridge tissue defects rather than replace an entire organ, typically ranging from a few millimetres to several centimetres, depending on the target tissue and defect geometry. However, its relatively low resolution (>300 µm) and potential for shear-induced cell damage present ongoing challenges [90]. Jetting-based bioprinting, in contrast, enables high-resolution (approximately 100 µm) and rapid patterning of low-viscosity materials, allowing precise spatial control over cells and biomolecules. However, its limited material range and challenges in fabricating thick 3D structures restrict its standalone applicability [14]. Vat photopolymerization-based bioprinting offers the highest geometric precision and fine architectural control (about 30 µm) through light-mediated crosslinking but remains constrained by the requirement for photo-crosslinkable bio-resins [77]. Hence, it is important to select an appropriate bioprinting technique based on its advantages, limitations and applications (Table 1) [11], [14], [77].

**Table 1:** Comparative table highlighting the advantages, limitations and applications of each bioprinting technique.

**Key characteristics**	**Extrusion-based**	**Jetting-based**	**Vat photopolymerization-based**	**References**
Typical resolution	>300 µm	Approximately 100 µm	Approximately 30 µm	[11], [14], [77]
Key advantages	Most widely adopted and versatile technique; Capable of printing high-viscosity, cell-laden bio-inks; Enables fabrication of large, mechanically robust, and clinically relevant-sized constructs; Compatible with multi-material printing	High resolution and patterning precision; Enables non-contact printing; Excellent control over the spatial distribution of cells and biomolecules	Highest spatial precision and surface finish; Enables fabrication of complex and fine three-dimensional (3D) architectures; Superior reproducibility and structural fidelity	[11], [14], [77]
Main limitations	Lower resolution compared to other techniques; Shear-induced cell damage from high printing pressure; Limited ability to achieve microscale architectural precision	Restricted to low-viscosity inks; Susceptible to nozzle clogging and droplet coalescence; Limited capability for fabricating thick 3D structures (< few mm); Lower throughput for large-scale constructs	Requires photo-crosslinkable bio-inks; Potential phototoxicity and reactive oxygen species (ROS) formation; Optical scattering limits printing depth; High system cost and limited material compatibility	[11], [14], [77]
Representative applications	Implantable, clinically relevant-sized constructs (e.g., bone, cartilage, muscle)	Thin tissue models (e.g., skin, alveolar lung)	Microstructured tissues (e.g., hepatic, microvascular)	[11], [14], [77]

No single approach currently fulfils the diverse requirements of functional tissue fabrication. Future progress will likely depend on multi-modal bioprinting strategies. Coupled with advances in bio-ink engineering, computational design, and real-time process monitoring, such integrative approaches hold the greatest promise for achieving complex, clinically translatable tissue constructs.

## 3 Bio-inks

The development of suitable bio-inks lies at the core of bioprinting, as these formulations not only determine the printability and fidelity of fabricated constructs but also govern cell viability, functionality, and long-term tissue maturation [91]. A bio-ink is a biocompatible hydrogel matrix that encapsulate living cells, biomolecules, and other functional components for precise spatial organization. Common bio-inks include natural polymers such as alginate, collagen, gelatin, fibrin, and decellularized extracellular matrix (dECM), which offer excellent biocompatibility and bioactivity, as well as synthetic polymers like poly(ethylene glycol) (PEG), Pluronic F127, and PVA, which provide tunable mechanical and rheological properties [92]. The performance of a bio-ink directly influences not only the printability and structural fidelity of the fabricated construct but also cell viability, functionality, and long-term tissue development [93]. Cell viability, commonly assessed through live/dead cytotoxicity assays where viable and non-viable cells emit green and red fluorescence, respectively, remains a key indicator of bio-ink compatibility, while metabolic assays such as AlamarBlue/PrestoBlue® or cell counting kit-8 (CCK-8) quantify cellular proliferation post-printing [94]. Importantly, bio-inks must satisfy a delicate balance of requirements: exhibiting favourable rheological properties for smooth extrusion or jetting, providing a supportive biochemical environment for cells, and possessing appropriate mechanical stability and degradation kinetics suited to the target tissue [95]. These design criteria often vary across different bioprinting modalities (extrusion-based, jetting-based, and vat photopolymerization-based systems), necessitating modality-specific optimization of bio-ink formulations.

### 3.1 Bio-inks for extrusion-based bioprinting

In extrusion-based bioprinting, cell-laden filaments are deposited in a continuous manner, imposing stringent requirements on bio-ink rheology. Formulations should exhibit shear-thinning behaviour, whereby viscosity decreases under shear during extrusion, enabling low-pressure flow through nozzles while minimizing shear-induced cell damage [96]. Once extruded, the bio-ink should rapidly regain its solid-like state to preserve structural fidelity, often stabilized by secondary crosslinking mechanisms.

Crosslinking plays a pivotal role in tuning both the printability and biological performance of extruded bio-inks [97]. Photo-crosslinking, a form of chemical crosslinking, introduces permanent covalent bonds through light-activated polymerization of (meth)acrylate-functionalized polymers such as GelMA [98], [99] or PEG-diacrylate (PEGDA) [100]. Advanced chemistries, such as thiol-ene click reactions, provide more homogeneous networks with reduced cytotoxic by-products [101], while enzymatic crosslinking using transglutaminase or horseradish peroxidase offers cytocompatible alternatives for protein- and peptide-based bio-inks [102], [103]. Physical crosslinking, relying on reversible interactions such as ionic bonding, hydrogen bonding, or hydrophobic forces, supports widely used systems such as alginate, carrageenan, chitosan, and gellan gum [104], [105], [106], [107].

More sophisticated supramolecular approaches such as host-guest complexation [108], DNA or peptide-based pairing [109], [110], and nanoparticle-reinforced formulations [111], [112] enhance mechanical robustness and enable dynamic, stimuli-responsive behaviours. Despite these advances, single-component hydrogels rarely achieve the dual requirements of high shape ﬁdelity and biofunctionality [11]. Interpenetrating network (IPN) hydrogels, incorporating both covalent and reversible crosslinks, improve structural integrity without sacrificing cell compatibility [113]. Pore-forming [114], [115] and microgel-based bio-inks [116], [117] further enhance cell viability by introducing interconnected porosity, facilitating nutrient and oxygen diffusion. Another promising approach involves dECM bio-inks, derived from specific tissues to recapitulate native biochemical and biophysical cues, thereby promoting cell-specific functionality [118]. Together, these innovations have significantly advanced the performance of extrusion-based bio-inks, although balancing mechanical demands with biological complexity remains an ongoing challenge.

### 3.2 Bio-inks for jetting-based bioprinting

Jetting-based bioprinting relies on the precise ejection of picolitre- to nanolitre-scale droplets, demanding bio-inks with tightly controlled physical properties [14]. Successful droplet formation is governed by a delicate interplay of surface tension, inertia, and viscosity, characterized by dimensionless parameters such as the Reynolds (Re), Weber (We), and *Oh* numbers [14]. These define a narrow operational window: viscosity should be low enough to permit droplet detachment (*Oh*<1), yet not so low as to induce jet instability and satellite droplets (*Oh*>0.1) [119]. As such, low-concentration polymeric solutions such as collagen [120], [121], [122], [123], GelMA [124], [125], or PEG derivatives [126], [127], [128] are commonly employed as bio-inks, balancing droplet formation with cell compatibility.

The incorporation of living cells within these bio-inks adds further constraints. Many cell-laden formulations exhibit non-Newtonian behaviour, while sedimentation of suspended cells in reservoirs leads to inconsistent cell density, nozzle clogging, and variability in droplet composition [129]. Strategies to mitigate these issues include adjusting formulation density to achieve neutral buoyancy [129], modifying the interior surface of printing cartridges to minimize cell adhesion arising from van der Waals interactions between cells and the cartridge wall [129], and applying active stirring [130]; however, each approach introduces trade-offs, particularly involving shear-sensitive cell types [131].

Despite these challenges, jetting-based bioprinting offers unparalleled spatial precision, enabling single-cell-level patterning of complex multi-cellular architectures [14]. This capability is critical for replicating native tissue microarchitectures, where cell positioning dictates key biological processes such as cell-cell interactions and paracrine signalling. Recent advances emphasize not only optimizing bio-ink rheology but also controlling droplet dynamics, such as controlling droplet velocities and minimizing splashing upon impact, to enhance deposition fidelity and cell viability [55], [56], [57]. Looking ahead, broadening the printability window of bio-inks, particularly for shear-sensitive and multi-cellular systems, will be essential to fully exploit the potential of jetting-based bioprinting in constructing biologically functional tissues.

### 3.3 Bio-inks for vat photopolymerization-based bioprinting

Vat photopolymerization-based bioprinting leverages photo-crosslinkable bio-resins that are selectively polymerized with light exposure, achieving microscale resolutions beyond the capabilities of extrusion-based or jetting-based approaches [132]. These bio-resins are typically composed of three key components: 1) monomers or macromers forming the structural hydrogel network, 2) PIs that generate radicals to trigger crosslinking, and 3) PAs that regulate light penetration to refine vertical (Z-axis) resolution [133]. The intricate balance among these components underpins the ability of vat photopolymerization-based systems to fabricate complex, high-fidelity constructs such as vascular-like networks and compartmentalized tissue models that critically influence cell behaviour.

The macromer forms the backbone of bio-resins and dictates the mechanical, degradative, and biological characteristics of the bioprinted constructs [77]. Photo-crosslinkable macromers such as GelMA, hyaluronic acid methacrylate (HAMA), and methacrylated silk fibroin are widely used due to their intrinsic bioactivity and cellular affinity [92]. However, these materials often require modification to optimize crosslinking kinetics and mechanical integrity, particularly for load-bearing or perfusable constructs [92]. Synthetic polymers such as PEGDA and polycaprolactone diacrylate (PCLDA) offer superior mechanical strength, predictable degradation profiles, and consistent quality, but lack intrinsic bioactivity, typically requiring functionalization with adhesion ligands or bioactive peptides [134]. Balancing these trade-offs remains a persistent challenge, compounded by vat photopolymerization-specific requirements such as low viscosity for efficient layer recoating and high optical transparency for uniform light penetration.

PIs are pivotal in vat photopolymerization-based bioprinting, as their absorption characteristics and radical-generation efficiency govern print speed, resolution, and cell viability [77]. Ideal PIs must exhibit strong absorption at the printer’s emission wavelength, high water solubility, and minimal cytotoxicity. Traditional ultraviolet (UV)-responsive PIs such as Irgacure 2959 (Type I) have been widely adopted due to their relatively low cytotoxicity but their poor water solubility and reliance on UV exposure limit applicability in cell-laden printing [135]. More recently, visible-light-responsive phosphine oxide-based PIs, including LAP and sodium diphenyl-2,4,6-trimethylbenzoylphosphinate (Na-TPO), have gained prominence due to their high reactivity, water solubility, and reduced cytotoxicity, enabling up to tenfold faster gelation compared to Irgacure 2959 in PEGDA-based hydrogels [136]. Alternative visible-light PIs systems leveraging naturally derived molecules such as riboflavin (vitamin B2) [137] or dye-based initiators such as eosin Y [138] offer enhanced biocompatibility, while metal complexes such as Ru(bpy)_3_^2+^ [tris(2,2’-bipyridyl) dichloro-ruthenium(II)], often used with co-initiators like sodium persulfate, provide additional tunability [139]. Despite these advances, each PIs system involves trade-offs in wavelength compatibility, reactivity, and cytocompatibility, highlighting the need for application-specific optimization.

PAs complement PIs by regulating light penetration to improve Z-axis resolution and prevent unintended polymerization outside the focal plane [140], [141]. Effective PAs should align their absorbance with the printer’s emission wavelength (e.g., 365 nm, 405 nm, 520 nm) while maintaining biocompatibility. Food-grade dyes such as tartrazine, curcumin, anthocyanins, acid red, and phenol red have been widely employed due to their water solubility and ease of removal [142], with curcumin offering additional radical-scavenging benefits [143]. Nanoparticle-based PAs, such as gold nanoparticles, provide tunable optical properties [144], while reactive PAs (e.g., diarylethene [145] or ketocoumarin derivatives [146]) integrate light attenuation with radical regeneration, enhancing both resolution and curing efficiency. However, excessive attenuation, leaching, or interference with cellular activity can compromise the photo-crosslinking mechanism.

The vat photopolymerization-based bioprinting is constrained by a narrow operational window. High PIs concentrations or increased light intensities improve structural fidelity but risk phototoxicity, while insufficient crosslinking can compromise mechanical integrity and long-term function [140], [141]. Furthermore, the predominantly single-vat configuration of current vat photopolymerization platforms limits multi-material printing without substantial hardware modifications [147]. Recent advances seek to address these limitations through strategies such as vat switching, vat-less photopolymerization, microfluidic bio-resin exchange, and multi-wavelength photopolymerization to enable complex, multi-material constructs [77]. Looking forward, research is expected to prioritize the development of water-soluble, visible-light PIs with minimal cytotoxicity; initiator-free or oxygen-tolerant crosslinking systems; multifunctional PAs that couple resolution enhancement with polymerization control; and composite bio-resins that combine the bioactivity of natural polymers with the tunability of synthetic analogues. These innovations promise to broaden the material design landscape, mitigate cytotoxic constraints, and fully unlock the potential of vat photopolymerization bioprinting for fabrication of structurally and biologically complex tissue constructs that closely recapitulate native microarchitectures.

### 3.4 Perspective on bio-ink development

The evolution of bio-ink design is closely intertwined with the technological requirements of extrusion-based, jetting-based, and vat photopolymerization-based bioprinting modalities. Each technique imposes distinct physicochemical and biological constraints on bio-ink formulations, thereby shaping their material composition, rheology, and crosslinking strategies.

Extrusion-based bioprinting demands bio-inks with shear-thinning and viscoelastic properties to facilitate continuous filament extrusion and rapid structural recovery post-deposition [96]. These requirements have driven the widespread use of multi-component hydrogels such as alginate, gelatin, and gellan gum stabilized through chemical, enzymatic, or physical crosslinking to enhance print fidelity and mechanical stability [97]. Composite formulations incorporating nanomaterials [111], [112], interpenetrating networks [113], or supramolecular linkages [148] further reinforce structural robustness and enable dynamic, stimuli-responsive behavior. However, extrusion bio-inks face a persistent trade-off between mechanical integrity and cell viability due to shear-induced stress during printing.

Jetting-based bioprinting operates on fundamentally different principles, relying on the ejection of picoliter-scale droplets under high-frequency actuation [14]. This technique requires low-viscosity bio-inks to achieve stable droplet formation while maintaining high cell viability. Common formulations include dilute polymeric solutions of collagen [120], [121], [122], [123], GelMA [124], [125], or PEG derivatives [126], [127], [128]. Despite offering exceptional spatial precision and cell patterning capabilities, jetting bio-inks are limited by their narrow operational viscosity window and susceptibility to nozzle clogging, particularly when incorporating high cell densities or particulate additives. Strategies such as neutral buoyancy adjustment [129], cartridge surface modification [129], or active stirring [130] partially mitigate these challenges, yet achieving consistent droplet uniformity across diverse cell types remains a key bottleneck.

Vat photopolymerization-based bioprinting employs photo-crosslinkable bio-inks polymerized by light exposure to achieve microscale resolutions and smooth surface topographies [18]. These photo-crosslinkable bio-inks typically consist of three major components: macromers (e.g., GelMA, HAMA, PEGDA) [92], [134], PIs [e.g., Irgacure 2959, LAP, Na-TPO] [135], [136], and PAs that regulate light penetration and Z-axis precision [140], [141]. This method excels in architectural precision but requires tight control over viscosity, optical transparency, and crosslinking kinetics to balance print resolution with cytocompatibility. While visible-light initiators and naturally derived absorbers have significantly improved biocompatibility, issues such as phototoxicity, single-material constraints, and limited multi-material integration continue to hinder clinical scalability.

Across these modalities, the central challenge lies in integrating engineering performance (printability, resolution, mechanical strength) with biological functionality (cytocompatibility, bioactivity, and tissue maturation potential). To this end, bio-inks are increasingly being categorized not only by their printing compatibility but also by their functional class: 1) naturally-derived bio-inks (e.g., collagen, gelatin, fibrin, hyaluronic acid, dECM) provide intrinsic biochemical cues and support cell adhesion and differentiation but often suffer from poor mechanical stability and batch variability [149]; 2) synthetic bio-inks (e.g., PEG, poly(ε-caprolactone) (PCL), Pluronic F127, PVA) offer tunable mechanical properties and reproducibility yet lack inherent bioactivity, necessitating chemical modification with peptides or growth factors [150]; 3) dynamic and stimuli-responsive bio-inks that are capable of responding to pH, temperature, or enzymatic activity, enable adaptive mechanical or biochemical changes that mimic native tissue remodelling [151]; and 4) dECM-based bio-inks are increasingly favoured for their tissue specificity, supporting cell-type dependent morphogenesis and maturation, though their rheological variability and limited scalability remain obstacles [152].

Emerging trends in bio-ink development are steering toward intelligent, multi-functional materials that act as active regulators of cell fate rather than passive scaffolds. These include self-healing and mechanoresponsive hydrogels, bio-inks with embedded biochemical gradients, and modular systems integrating organoid or spheroid assemblies for prevascularization and tissue maturation. Concurrently, coupling advanced material chemistries with computational modeling, rheological simulation, and insitu monitoring enables real-time optimization of print parameters and structural fidelity. In the future, the convergence of material science, biophysics, and biofabrication technologies will define the next generation of bio-inks that can support long-term cell viability, guide tissue morphogenesis, and ensure reproducibility across platforms. Such integrative design approaches will be pivotal in realizing clinically viable, adaptive, and functionally mature bioprinted tissues, bridging the current gap between laboratory prototypes and therapeutic implementation.

## 4 3D bioprinting strategies for modelling and treating systemic diseases

A systemic disease is a medical condition that affects the body as a whole or involves multiple organs and tissues, rather than being localized to a specific area. Such diseases often manifest with diverse symptoms and can compromise different physiological systems simultaneously. Examples include: 1) cardiovascular diseases such as hypertension, atherosclerosis, and heart failure, 2) metabolic and endocrine diseases such as diabetes mellitus and thyroid disorders, and 3) neurodegenerative diseases such as Alzheimer’s disease and Parkinson’s disease. Addressing systemic diseases poses a major challenge due to their multi-organ involvement and complex pathophysiology.

3D bioprinting is uniquely positioned to advance research and treatment in this domain. It enables the fabrication of disease-relevant human tissues with controlled architecture and cellular composition, the creation of vascularized and perfusable constructs that recapitulate hemodynamics and barrier functions, and the development of modular multi-tissue platforms that reproduce organ-organ interactions. By bridging the gap between *in vitro* modelling and *in vivo* physiology, bioprinting provides powerful opportunities for mechanistic studies, drug discovery, and regenerative therapies. This section links key systemic diseases to the bioprinted tissues and functionalities required to investigate and ultimately address them.

### 4.1 Cardiovascular tissue bioprinting

Cardiovascular diseases remain the leading cause of morbidity and mortality worldwide, encompassing conditions that affect the heart and vasculature, such as arrhythmias, coronary artery disease, cardiomyopathies, heart failure and valvular disorders [153]. These diseases often involve progressive structural and functional deterioration of cardiac tissue, limited intrinsic regenerative capacity, and irreversible injury following myocardial infarction [153]. Conventional treatment strategies, including pharmacological management, interventional procedures, and organ transplantation, can alleviate symptoms or slow disease progression, but rarely restore native cardiac function [154]. Moreover, the persistent shortage of donor hearts highlights the urgent need for alternative therapeutic solutions [154].

The heart, a muscular organ essential for sustaining life, comprises 4 distinct layers: the protective pericardium, the epicardium, the thick contractile myocardium, and the innermost endocardium [155]. Reproducing this highly specialized, multicellular architecture, characterized by anisotropic alignment, electrical conductivity, and functional contractility, remains a major challenge in cardiac tissue engineering.

**Fig. 2:**
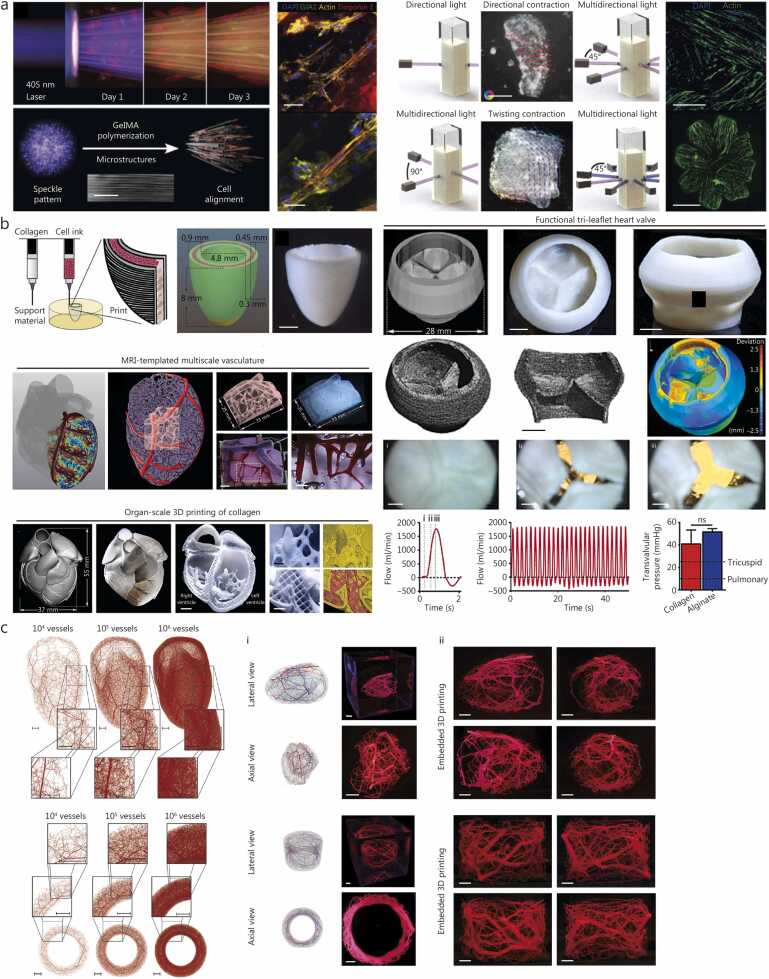
3D bioprinting strategies for cardiovascular diseases. **a** Filamented light biofabrication, where cell-induced light scattering and bio-resin crosslinking generate aligned cardiomyocytes (red) and fibroblasts (yellow) (scale bar=25 μm, 50 μm, 200 μm, 500 μm, and 1 mm, respectively). This enables multi-directional alignment and torsional contraction in induced pluripotent stem cell-cardiomyocytes cardiac constructs, as confirmed by immunostaining and optical flow analysis. Reproduced with permission, licensed under CC-BY-4.0 [88]. **b** Dual-material support-bath bioprinting using collagen and high-concentration cellular bio-inks for ventricle fabrication, showing layered cardiac cells (pink), collagen shells (green), and collagen-only regions (yellow); along with micrograph of bioprinted ventricle. This strategy supports the fabrication of diverse human heart components using collagen bio-inks, from fine capillary structures to full organ-scales (scale bars are 2 mm and 5 mm). Doppler flow velocimetry of a single cycle: (i) closed, (ii) half-open, and (iii) open. Reproduced with permission [29]. **c** Algorithmic design and bioprinting of scalable vascular networks across terminal vessel densities (10^4^–10^6^), demonstrated in biventricular and annulus geometries (scale bar=1 cm). Printed models (Carbopol with red pigment) show interconnected branches and orthogonal views of vascularized tissues. Reproduced with permission [164]. GelMA. Gelatin methacryloyl; ns. Not significant; MRI. Magnetic resonance imaging; 3D. Three-dimensional.

3D bioprinting has emerged as a transformative tool to overcome these challenges by enabling the precise fabrication of cardiac tissues with spatially organized architecture and defined cell deposition [156]. Among the various bioprinting modalities, vat photopolymerization-based bioprinting methods have been particularly instrumental in advancing high structural fidelity. A notable example is FLight bioprinting, which exploits optical modulation instability to generate highly aligned microfilament networks with tunable resolutions of 2–30 µm through control of the light beam’s coherence length [88]. Using this approach, researchers fabricated high-density cardiac tissues [(1.5–6.0)×10^7^ cells/ml] with controlled multi-directional alignment, including tri-layered myocardium and bi-layered constructs, composed of human induced pluripotent stem cells (hiPSCs)-derived cardiomyocytes (Fig. 2**a**). To mitigate cell-induced light scattering during photopolymerization, a refractive index-matching solution (40% w/v iodixanol) was combined with 5% w/v GelMA, enabling the formulation of high-cell-density bio-inks. Compared with unstructured controls, these engineered tissues (2.0 mm×2.0 mm×0.5 mm) exhibited elongated, striated morphology, confirmed by actin and troponin 1 staining, and enhanced intercellular connectivity, as indicated by increased expression of gap junction protein connexin-4 [157]. Such microscale bioprinted constructs are particularly valuable for drug screening and disease modelling, where reproducibility, miniaturization, and responsiveness to pharmacological stimuli are prioritized over long-term vascularization or large-scale contractility. By contrast, bioprinted tissues for transplantation must achieve greater structural and physiological complexity, incorporating multicellular organization, pre-vascular networks, and the mechanical robustness required for surgical handling and integration with host myocardium [158].

Extrusion-based bioprinting complements these approaches by enabling the fabrication of larger, high cell-density cardiac constructs derived from hiPSCs-derived cardiomyocytes that progressively mature into spontaneously beating and synchronized tissues [159], [160], [161]. For example, support-bath extrusion bioprinting has been used to print geometrically complex, anatomically-relevant structures. A recent study employed a complex coacervation method to generate gelatin microgels with uniform morphology, reduced particle size (approximately 25 µm), and low polydispersity [29]. Using this platform, researchers bioprinted a range of cardiac components, from capillary-scale features to organ-level scale constructs (Fig. 2**b**). The resulting 3D-bioprinted cardiac ventricle, designed as an open ellipsoidal shell (6.6 mm maximum outer diameter, 8.0 mm length from base to apex), was composed of human embryonic stem cell (hESC)-derived human cardiomyocytes (3.0×10^8^ cells/ml) and 2% human ventricular cardiac fibroblasts. The bioprinted ventricle recapitulated patient-specific cardiac geometry and exhibited advanced physiological performance, including synchronized contractions responsive to external pacing, directional excitation wave propagation (observed by calcium transient mapping), and dynamic wall thickening during peak systole [29].

Beyond myocardial tissues, fabricating integrated vascular networks remains essential for the long-term viability of transplantable constructs, as these networks facilitate nutrient delivery, perfusion, and functional maturation. Emerging strategies include support-bath bioprinting of sacrificial channels [160] and co-axial extrusion of hollow, perfusable tubular structures [162], [163]. A notable advance involved the rapid extrusion-based bioprinting of organ-spanning vascular tree networks within a 90 mm×90 mm×90 mm support bath using human embryonic kidney 293 cell-laden bio-inks (1.0×10^7^ cells/ml) within minutes, integrating computational hemodynamic simulations and automated design-to-print workflows (Fig. 2**c**) [164]. Despite this progress, challenges remain in achieving fine-scale vascular fidelity and maintaining perfusion continuity within thick tissues.

3D bioprinting offers a continuum of capabilities ranging from miniaturized cardiac microtissues for high-throughput drug testing to macroscale, vascularized constructs for regenerative transplantation. While most current research emphasizes thin or simplified cardiac tissues, future efforts should integrate advances in biomaterial design, vascularization, and accelerated tissue maturation (electromechanical conditioning [165], [166]) to bridge the gap between *in vitro* functional models and *in vivo* therapeutic applications.

### 4.2 Endocrine and metabolic tissue bioprinting

Endocrine and metabolic diseases, including diabetes mellitus, chronic liver disease, and chronic kidney disease, represent a major global health burden, affecting hundreds of millions worldwide [167]. These disorders arise from complex interactions among genetic, environmental, and lifestyle factors that progressively disrupt systemic metabolic homeostasis [168]. The pancreas, liver, and kidney form the core regulatory systems of endocrine and metabolic control, yet each exhibits distinct structural and functional architectures from the hormone-secreting islets of Langerhans to the detoxifying hepatic lobules and the filtration units of the nephron [168]. Replicating these organ-specific microarchitectures and functions has been a longstanding challenge for conventional tissue engineering. Recent advances in 3D bioprinting now enable the fabrication of physiologically relevant constructs for both drug screening and transplantation, although these two application domains differ significantly in their design goals, material composition, and functional assessment criteria.

#### 4.2.1 Pancreatic tissue constructs

Pancreatic islets are compact clusters of endocrine cells that coordinate glucose homeostasis through insulin and glucagon secretion. Conventional stem cell-derived islets often remain immature and exhibit suboptimal glucose-stimulated insulin secretion (GSIS) [169]. 3D bioprinting enables the spatial organization of β-cells, stromal, and endothelial components to enhance maturation and function.

For screening applications, bioprinted islet constructs are designed for high-throughput GSIS quantification and cytokine profiling. For instance, extrusion-based bioprinting of primary islets with immunomodulatory adipose-derived stromal cells (1.4×10^7^ cells/ml) in alginate/nanofibrillated cellulose bio-inks produced double-layered scaffolds (10 mm×10 mm×1 mm) that enhanced GSIS quantified via insulin enzyme-linked immunosorbent assay (ELISA), and reduced inflammatory cytokine secretion such as monocyte chemoattractant protein-1 (MCP-1), interferon gamma-induced protein-10 (IP-10), and growth-regulated protein-α (GRO-α), quantified using multiplex immunoassay [170].

For transplantation models, structural integrity, vascular integration, and long-term glycemic regulation are prioritized. DLP-bioprinted islet organoids (2500 islet equivalent/ml, approximately 150 µm per islet, 10 mm diameter×2 mm thick) using HAMA/pancreatic dECM bio-inks and primary murine islets achieved prolonged normoglycemia for up to 90 d post-implantation, with ras-related C3 botulinum toxin substrate 1 (Rac1)/rho-associated coiled-coil containing protein kinase (ROCK)/myosin light chain kinase (MLCK)-mediated neovascularization confirmed via Western blotting [171]. Recent support-bath extrusion-based bioprinting of hESC-derived islet cells and human umbilical vein endothelial cells (HUVECs) (5:1 ratio, 1.0×10^8^ cells/ml) produced vascularized human islet-like cellular aggregate (HICA, about 300 µm diameter) with improved islet morphogenesis [expression of connexins (CX36, CX43) and vascular endothelial cadherin (VE-CAD)], and stable glucose responsiveness under diabetic conditions [172].

#### 4.2.2 Hepatic tissue constructs

Bioprinted hepatic tissues are developed for drug metabolism studies and liver regenerative therapy, each requiring distinct structural and functional outcomes; while drug-screening models prioritize microscale fidelity, high-throughput fabrication, and reproducible metabolic activity for assessing hepatotoxicity and enzyme kinetics, transplantation-oriented constructs must instead achieve macroscale organization, perfusable vasculature, and long-term functional integration with host circulation to restore hepatic physiology [173], [174].

For drug-screening platforms, small-scale, high-throughput liver constructs are designed to replicate hepatocellular metabolism and xenobiotic responses. DLP bioprinting of hexagonal lobule-like constructs containing hepatocytes, endothelial, and stromal cells successfully reproduced liver-specific functions, including albumin secretion, cytochrome P450 enzyme activity, and bile canaliculi formation (assayed via ELISA and immunofluorescence) [173], [174]. Incorporating liver dECM bio-inks improved viability and upregulated hepatic gene expression [reverse transcription quantitative polymerase chain reaction (RT-qPCR)] [175], [176]. Similarly, extrusion bioprinting of human-induced hepatocytes (hiHeps, 1.0×10^6^ cells/ml) yielded cost-effective models (∅ 10 mm, 2 mm height), showing robust metabolic activity (validated by albumin, cytochrome P450 activity measurements) and dose-dependent cytotoxicity responses to acetaminophen [177]. These platforms thus provide physiologically relevant tools for personalized toxicity testing and pharmacokinetic modelling.

For transplantation models, constructs should achieve mechanical robustness, perfusability, and sustained metabolic function *in vivo*. Integration of human chemically induced pluripotent stem cells (hCiPSCs)-derived hepatocyte organoids (1.5×10^7^ cells/ml) into extrusion-based bioprinted scaffolds improved hepatic survival and regeneration in acute and chronic failure models [178]. Perfusable, vascularized constructs fabricated via extrusion-based bioprinting using HepG2-laden microgel-hydrogel hybrids (1.0×10^8^ cells/ml) demonstrated host vascular integration [CD31 and α-smooth muscle actin (α-SMA) immunostaining] and maintained *in vivo* albumin secretion and reduced serum transaminases [alanine aminotransferase (ALT) and aspartate aminotransferase (AST)] after transplantation [179].

#### 4.2.3 Renal tissue constructs

The kidney’s complex microarchitecture presents distinct design constraints for *in vitro* screening versus implantable renal tissues [180]; while screening constructs focus on mimicking localized nephron segments to study filtration, reabsorption, and fibrosis under controlled microfluidic or biochemical stimuli [181], transplantable constructs demand hierarchical organization with interconnected glomerular, tubular, and vascular compartments to sustain fluid exchange and mechanical stability under physiological pressures [182], [183].

For drug-screening models, the primary goal is to replicate nephron-specific filtration and reabsorption within perfusable microenvironments [181]. Bioprinted renal tubulointerstitium-on-chip models containing primary human renal proximal tubular epithelial cells and primary human kidney fibroblast cells (each 8.0×10^6^ cells/ml) were fabricated via extrusion-based bioprinting to model fibrosis through modulation of matrix stiffness and transforming growth factor (TGF)-β1 exposure. The renal fibrosis can be determined using functional assays, including RT-qPCR quantification of fibrotic genes [actin, alpha 2, smooth muscle (*ACTA2)*, fibronectin 1 (*FN1)*, vimentin (*VIM)*, collagen, type I, alpha 1 chain (*COL1A1)*, collagen, type III, alpha 1 chain *(COL3A1)*, collagen, type IV, alpha 1 chain (*COL4A1*)], collagen staining (from histology sections stained for picrosirius red), and protein deposition (BCA protein assay) [181]. Such platforms provide clinically relevant alternatives to animal models, reducing reliance on *in vivo* studies and thereby aligning with ethical principles in biomedical research. They enable mechanistic studies and drug screening while minimizing ethical concerns associated with animal experimentation.

In contrast, transplantable renal constructs prioritize structural complexity and vascularization. A recent study demonstrated extrusion-based bioprinting with photo-crosslinkable kidney dECM methacrylate (KdECMMA) bio-inks containing human primary kidney cells (followed by UV-induced crosslinking) to produce renal-like constructs (8 mm×4 mm×1.6 mm) yielded glomerular- and tubular-like regions confirmed by immunostaining for nephrotic syndrome type 2 (NPHS2), aquaporin 1 (AQP1), and paired box gene 2 (PAX2) after implantation in athymic RNU rats [182]. Another study demonstrated coaxial extrusion-based bioprinting of dECM/alginate bio-inks loaded with HUVECs (1.0×10^7^ cells/ml) and renal proximal tubular epithelial cells (2.0×10^7^ cells/ml) to produce hollow renal tubules capable of albumin reabsorption and selective permeability, validated by fluorescein isothiocyanate (FITC)-albumin transport assays and tight-junction staining [183].

3D bioprinting is transforming the engineering of endocrine and metabolic tissues by enabling both high-throughput screening models and implantable regenerative tissues. Screening constructs emphasize miniaturization, reproducibility, and assay compatibility, typically validated through metabolic activity (GSIS, albumin, CYP450, urea, or reabsorption assays) and cytotoxicity readouts. In contrast, transplantation models require macroscale architecture, perfusability, and long-term functionality, validated by vascularization (CD31, VE-CAD), integration (histology, immunostaining), and *in vivo* metabolic restoration. Achieving reproducibility and scalability across both domains requires harmonizing bio-ink composition, cell sourcing, and standardized assay protocols. The integration of biomaterials science, stem cell biology, and advanced bioprinting thus positions this technology as a powerful and versatile platform for addressing diabetes, liver failure, and kidney disease.

### 4.3 Neurodegenerative tissue bioprinting

Neurodegenerative diseases such as Alzheimer’s disease and Parkinson’s disease present a major global health burden, characterized by progressive neuronal loss, synaptic dysfunction, and cognitive or motor decline [184]. Conventional 2D neuronal cultures and animal systems often fail to recapitulate the human central nervous system (CNS)’s spatial microarchitecture, cellular diversity, and long-range connectivity [185]. 3D bioprinting addresses these limitations by enabling the fabrication of physiologically relevant neural constructs that integrate neurons, glia, extracellular matrix (ECM) cues, and vascular networks within controlled 3D microenvironments. Depending on design intent, these constructs serve two complementary roles: screening models, optimized for mechanistic studies and neuropharmacological testing, and transplantation models, engineered for *in vivo* repair and functional recovery.

**Fig. 3:**
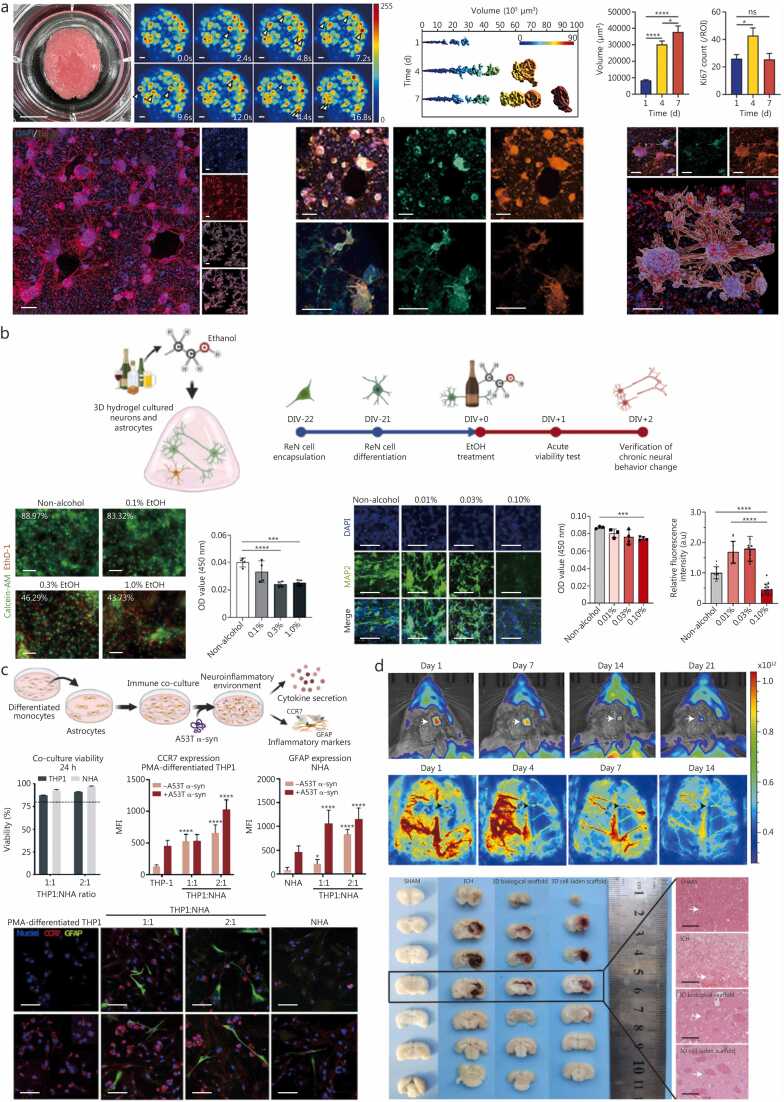
3D bioprinting strategies for neurodegenerative diseases. **a** 3D bioprinted brain tissue showing calcium transients (2.4 s intervals; white arrows indicating the onset of neuronal firing) and neural network architecture with cluster volumes quantified at 1, 4, and 7 d *in vitro* (*n*=4). Ki-67^+^ cell counts (from 10× magnification images) and immunofluorescence images of DAPI (blue), NeuN (green), MAP2 (red), phalloidin (green), GFAP (red), Nestin (green), and Tuj1 (red), with 3D reconstructions of clusters and neural networks. Scale bar=200 μm. Reproduced with permission, licensed under CC-BY-4.0 [187]. **b** Alcohol-induced neural alterations: schematic of alcoholic neural disorder, live/dead analysis (moderate alcohol exposure), mitochondrial activity using CCK assay (moderate/heavy exposure), and immunofluorescence quantification of neurogenesis under alcohol. Scale bars=50 μm and 100 μm. Reproduced with permission, licensed under CC-BY-4.0 [188]. **c** A53T α-syn-induced neuroinflammation model: PMA-differentiated THP-1 monocytes co-cultured with normal human astrocytes (NHA) at 1:1 or 2:1 ratio and stimulated with A53T α-syn (10 µg/ml). Viability (>80%) confirmed by live/dead assay. Elevated mean fluorescence intensity of proinflammatory markers CCR7 (monocytes) and GFAP (astrocytes) in co-culture conditions. Representative images of CCR7/GFAP in co-cultures (1:1, 2:1) and monocultures. Nuclei stained with Hoechst 332 (blue). Scale bar=70 μm. Reproduced with permission, licensed under CC-BY-4.0 [190]. **d***In vivo* tracking of luciferase-labelled scaffolds via bioluminescence imaging (0–21 d) showing (92.3±5.1)% signal loss by day 21, and H&E staining demonstrated scaffold-driven peri-haematomal vascular regeneration and perfusion recovery. Scale bar=100 μm. Reproduced with permission, licensed under CC-BY-4.0 [192]. 3D. Three-dimensional; DIV. Days *in vitro*; EtOH. Ethanol; ReN. ReNcell® (immortalized human neural progenitor cell lines); CCR7. C-C chemokine receptor type 7; GFAP. Glial fibrillary acidic protein; THP1. Human monocytic leukemia cell line; DAPI. 4′;6-diamidino-2-phenylindole; NeuN. Neuronal nuclear protein; MAP2. Microtubule-associated protein 2; CCK. Cell counting kit; PMA. Phorbol 12-mystrate 13-acetate; α-syn. α-synuclein; MFI. Mitochondrial functional index.

Screening models emphasize reproducible network formation, electrophysiological responsiveness, and readouts suitable for mechanistic or drug assays. To recapitulate functional neural circuits, hyaluronic acid/fibrinogen bio-inks have been used in extrusion-based bioprinting to assemble defined cortical and striatal neuronal subtypes co-printed with astrocyte progenitors at high cellular densities (1.0×10^7^ cells/ml), producing thin multilayered tissues (<50 µm thickness) that established synaptic connectivity and increased calcium responses within 2–5 weeks [186]. Similarly, GelMA-based neural tissue constructs (7.0 mm×7.0 mm×1.5 mm) fabricated via extrusion-based bioprinting with embryonic rat cortical neurons (1.5×10^7^ cells/ml) progressively developed local and long-range functional axon connections (assessed using Tuj1 immunostaining and Fluo-4 acetoxymethyl ester imaging) and showed similar transcriptomic profiles (quantified via RNA sequencing) to the cerebral cortex. When treated with oxygen-glucose deprivation/reperfusion, the developed neural tissue constructs displayed downregulation of pathways related to ligand-receptor interactions, gated channels, and synapse components, which is consistent with the neuronal damage caused by an ischemic stroke, making them suitable for ischemic stroke modelling (Fig. 3**a**) [187].

More complex engineered neural networks have been produced using extrusion-based bioprinting of brain-derived dECM bio-inks with human neural progenitor cells (NPCs) (5.0 × 10^6^ cells/ml) to fabricate larger neural tissue constructs (10.0 mm × 10.0 mm × 0.2 mm) that displayed mature neuronal morphology, spontaneous calcium signalling, and reliable axonal dynamics [188]. These neural models facilitated spatiotemporal mapping of degeneration such as exposure to ethanol-induced axonal deformation and amyloid-β formation that could be directly imaged in real time, thereby providing insights into region-specific neurotoxicity and disease onset (Fig. 3**b**). For disease-specific screening, extrusion-based bioprinted dome-shaped constructs (∅ 10 mm×5 mm) with hiPSCs-derived NPCs (1.0×10^6^ cells/ml) from Alzheimer’s disease patients differentiated into basal forebrain cholinergic neurons (via puromorphamine-releasing microspheres) and exhibited amyloid-β and tau deposition (quantified via immunocytochemistry analysis) alongside immature electrical activity after 30–45 d [189]. Similarly, Parkinson’s disease screening models (∅ 6 mm×2 mm) fabricated using extrusion-based bioprinting, characterized by dopaminergic neuron loss and neuroinflammation, were fabricated using methacryloyl-modified small intestinal submucosa dECM-derived bio-inks containing differentiated NPCs (1.0×10^7^ cells/ml) [190]. These dense dopaminergic networks, when co-cultured with seeded astrocytes [(1.50–2.25)×10^4^ cells/ml] and THP-1 macrophages (2.0×10^5^ cells/ml), reproduced neuroinflammatory responses to pathogenic A53T α-synuclein (α-syn) exposure (Fig. 3**c**). These screening platforms outperform 2D assays in capturing multi-cellular interactions and are well suited for mechanistic studies and medium-throughput therapeutic screening.

Transplantation models prioritize biocompatibility, structural stability, vascular integration, and behavioural or physiological recovery after implantation. Small, implantable dot constructs (∅ 1.00 mm×0.25 mm) printed from gelatin-alginate fibrinogen bio-inks containing hiPSCs-derived NPCs (3.5×10^6^ cells/ml) were shown to mimic brain elasticity while supporting long-term neurogenesis and gliogenesis *in vitro*; when transplanted into neonatal rat hippocampus, these patches demonstrated spatial stability and survival beyond one month, indicating potential for cell-based repair [191]. For acute CNS injury, human umbilical cord-derived mesenchymal stem cells (1.0×10^6^ cells/ml) encapsulated in gelatin/alginate/porcine brain dECM-based bio-inks were fabricated using extrusion-based bioprinting as 3D cell-laden scaffolds (2 mm×2 mm×1 mm) and implanted in rat models of intracerebral haemorrhage. Compared to conventional stem cell suspension therapies, 3D-bioprinted implants improved cell retention, promoted angiogenesis (CD31/α-SMA staining), and enhanced functional sensorimotor recovery (Fig. 3**d**), suggesting clinical potential for neuronal repair in stroke and haemorrhagic injury [192].

These studies illustrate a clear functional split: screening constructs are typically small (sub-mm to a few mm), highly cellular (10^6^–10^7^ cells/ml), and optimized for quantitative *in vitro* assays such as live calcium imaging, multielectrode-array recordings, and biochemical quantification of disease markers; whereas transplantation constructs are designed for *in vivo* stability, vascularization, and host integration, with are evaluated using histology and immunostaining for engraftment, endothelial markers (e.g., CD31) together with perfusion assays to demonstrate functional vascular integration, electrophysiological measurements, and behavioural recovery. Importantly, most transplantation studies remain constrained to small, rodent-scaled implants with incomplete recapitulation of higher-order cortical organization, hence, successful engraftment in rats does not directly predict human outcomes. Future work should therefore prioritize validation in larger animal or ex vivo human tissue platforms to bridge the translational gap from predictive screening models to clinically relevant regenerative therapies.

## 5 3D bioprinting strategies for repair and regeneration of localized tissue injuries

Localized tissue injuries including damage to the skin, muscle, cartilage, and bone are among the most frequent and debilitating conditions encountered in both civilian and battlefield environments. These injuries may result from trauma, burns, blast impacts, or degenerative diseases, leading to extensive tissue loss, compromised function, and long-term disability [193]. Conventional reconstructive interventions such as autografts, allografts, and prosthetic implants are often constrained by donor site morbidity, immune rejection, poor vascular integration, and limited ability to restore native tissue architecture [194]. 3D bioprinting has emerged as a transformative platform that enables the fabrication of patient-specific, biomimetic tissue constructs through precise deposition of living cells and biomaterials. By integrating multiple cell types, bioactive factors, and ECM-mimicking bio-inks, this technology allows for the engineering of tissue-specific microenvironments and mechanical anisotropy critical for functional regeneration [195].

The following sections outline major advances in 3D bioprinting strategies for localized tissue repair, progressing from skin (the body’s primary protective barrier) to more complex musculoskeletal, cartilage, and bone tissues. This organization reflects increasing structural and biological complexity, highlighting how advances in biomaterials, stem cell biology, and printing precision drive next-generation regenerative therapies.

### 5.1 Skin injuries

Skin possesses an intrinsic regenerative capacity mediated by keratinocytes (KCs) proliferation, fibroblasts (FBs)-driven ECM remodelling, and neovascularization. However, severe or deep wounds such as those caused by burns, chronic ulcers, or ballistic trauma often overwhelm these natural processes, leading to delayed wound closure, infection, and fibrotic scarring [196]. Traditional interventions such as split-thickness skin grafting remain limited by donor shortages, graft contraction, and insufficient vascularization [197]. 3D bioprinting provides an alternative approach. Enabling the generation of anatomically accurate, multi-layered skin constructs that replicate epidermal, dermal, and hypodermal architecture [198], [199]. Through spatially defined deposition of KCs, FBs, endothelial cells (ECs), and pericytes (PCs) within bio-inks, bioprinted skin can achieve functional and structural fidelity approaching that of native tissue.

One notable example demonstrated the fabrication of vascularized, xeno-free human skin constructs using primary human KCs, FBs, ECs, and PCs derived from a single neonatal donor [200]. ECs proliferation and barrier integrity were quantified via transendothelial electrical resistance (TEER), while endothelial responsiveness was confirmed through tumour necrosis factor-α (TNF-α) stimulation assays. The dermal compartment was bioprinted using a bio-ink comprising ECs (4.5×10^6^ cells/ml), FBs (4.5×10^6^ cells/ml), and PCs (5.5×10^6^ cells/ml) suspended in human collagen I (6 mg/ml), fibronectin (1 mg/ml), and VitroGel. This was printed into a 13 mm-diameter, 1 mm-thick polyglycolic acid (PGA) mesh, followed by deposition of an epidermal KCs layer (2.0×10^6^ cells/ml). Upon implantation into severe combined immunodeficient (SCID)/bg mice, the constructs developed perfused micro-vessels (UEA-I^+^/hCD31^+^) and stratified epidermis expressing CK10, CK14, laminin-5, and collagen IV within 2 weeks. Controlled PGA degradation and absence of chronic inflammation demonstrated stable graft integration and vascular perfusability.

Similarly, a plasma-derived fibrin bio-ink composed of fibrinogen (2.3 mg/ml), tranexamic acid (200 µl) and CaCl_2_ (1% w/v) was used to bioprint human skin equivalents containing FBs (1.75×10^4^ cells/ml) and KCs (6.0×10^6^ cells per P100 plate) [201]. After 17 d of air-liquid interface culture, histological and immunohistochemical analyses confirmed dermal (vimentin), epidermal (K5, K10, filaggrin), and basement membrane (collagen VII) markers, indicative of a stratified epidermis with mature rete ridges. Upon transplantation to full-thickness wounds (∅ 12 mm) in immunodeficient nude mice, the constructs achieved robust graft integration, vascularization (SMA⁺ vessels), and human cell persistence, highlighting translational potential for treating burns and chronic wounds.

To further enhance vascular integration, a prevascularized, multi-layered human skin construct was fabricated using a dermal bio-ink containing FBs (7.0×10^5^ cells/ml), ECs (7.0×10^5^ cells/ml), and PCs (3.5×10^5^ cells/ml) in collagen I (3.5 mg/ml), combined with an epidermal KCs bio-ink (2.0×10^6^ cells/ml) [202]. The constructs matured into vascularized tissues expressing CK14, CK10, filaggrin, CD31, and ulex europaeus agglutinin I (UEA-1), and upon implantation into full-thickness murine wounds, achieved host inosculation within four weeks. Notably, PCs incorporation improved vascular invasion and dermal-epidermal junction integrity, demonstrating feasibility of prevascularized “off-the-shelf” allogeneic skin grafts.

**Fig. 4:**
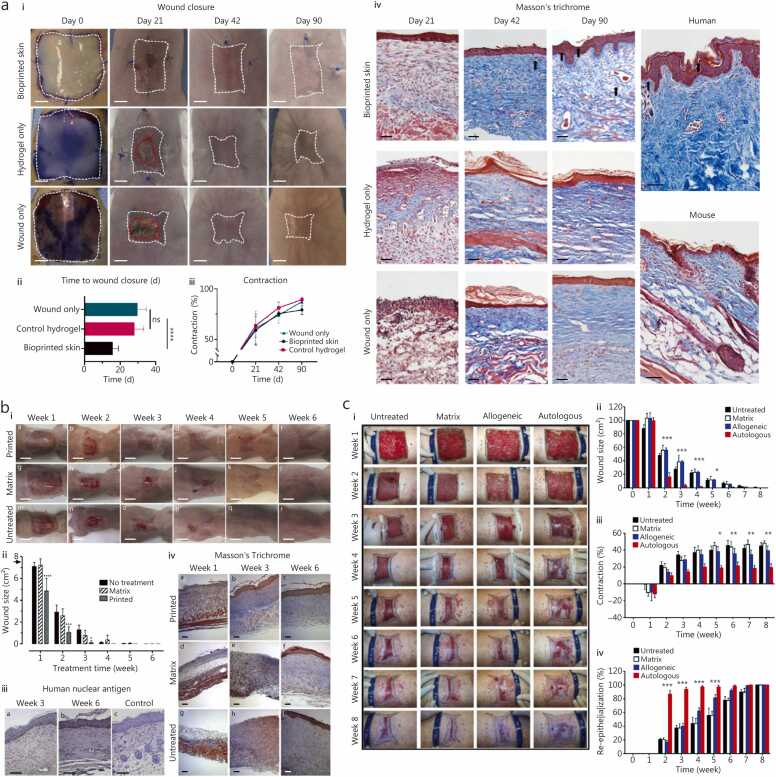
3D bioprinting strategies for skin repair. **a** Bioprinted human skin accelerates wound closure, improves epidermal barrier formation, and supports organized ECM remodelling. Representative images of i) bioprinted, hydrogel-only, and untreated murine full-thickness wounds (scale bar=5 mm), ii, iii) with planimetry for wound closure and contraction, iv) and Masson’s trichrome staining showing progressive matrix organization compared to controls (scale bar=50 μm). Reproduced with permission [203]. **b***In vivo* murine evaluation. (i) Results demonstrate rapid epithelialization, complete closure by week 3, and minimal contraction in printed-skin wounds, with delayed healing in matrix-only and untreated groups (scale bar=1 cm). Quantitative analysis confirms significantly faster closure (ii), persistent human cell engraftment (iii), and improved collagen organization (iv) (scale bar=100 μm). Reproduced with permission, licensed under CC-BY-4.0 [204]. **c***In-situ* bioprinting in a porcine model shows early epithelial island formation and full re-epithelialization with minimal contraction in autologous cell-treated wounds, whereas allogeneic, matrix-only, and untreated groups exhibit marked contraction and limited epithelialization. Reproduced with permission, licensed under CC-BY-4.0 [204]. 3D. Three-dimensional; ECM. Extracellular matrix.

Expanding on structural complexity, tri-layered human skin constructs (3 cm×3 cm) were fabricated using region-specific bio-inks comprising epidermal (KCs, melanocytes), dermal (FBs, follicle papillary cells, microvascular ECs), and hypodermal pre-adipocytes (20.0×10^6^ cells/ml each) [203]. Printed with a 500 µm nozzle in a fibrinogen matrix (30 mg/ml) crosslinked with thrombin (20 U/ml), the constructs maintained >75% viability and stable stratification for up to 52 d. Functional assays including live/dead staining, epidermal cornification, and immunostaining for KCs (KRT14, IVL), FBs (vimentin), vascular (CD31, CD146), and ECM (collagen I/III) markers, confirmed tissue maturation. When implanted in athymic mice and porcine models, the bioprinted constructs accelerated epithelialization, enhanced rete ridge formation, and reduced wound contraction, validating their potential as vascularized, full-thickness skin substitutes (Fig. 4**a**).

For point-of-care applications, a mobile *in-situ* bioprinting platform was developed using an XYZ robotic arm equipped with eight 260 µm inkjet nozzles and laser scanning for real-time wound mapping [204]. A fibrin/collagen matrix containing autologous FBs (3.75×10^6^ cells/ml) and KCs (7.5×10^6^ cells/ml) was directly bioprinted onto murine dorsal wounds (3.0 cm×2.5 cm), achieving complete wound closure within three weeks (Fig. 4**b**). In a porcine model with 10 cm×10 cm wounds, autologous cell bioprinting resulted in full re-epithelialization and formation of stratified epidermis with CD31^+^ vasculature and Ki-67^+^ proliferating KCs by week 5 (Fig. 4**c**). These findings highlight the clinical feasibility of rapid, on-demand bioprinting for personalized wound coverage, particularly relevant in military or emergency settings where immediate tissue replacement is critical.

These studies illustrate the continuum from *in vitro* engineered, vascularized skin to *in situ*, patient-specific bioprinting, highlighting its potential to revolutionize wound management. Skin bioprinting offers precise spatial control, reduced donor dependency, and scalability for extensive injuries. Nonetheless, challenges remain in replicating full innervation, appendage formation, and long-term mechanical stability, as well as ensuring sterility and robustness of portable bioprinting platforms. Moving forward, integration of advanced bio-ink formulations, vascular and neural network engineering, and adaptive real-time printing will be essential to translate these innovations into reliable, deployable solutions for both civilian healthcare and military medicine.

### 5.2 Musculoskeletal injuries

Skeletal muscle possesses a remarkable intrinsic regenerative capacity mediated by satellite cell activation following minor injuries [205]. However, when trauma exceeds a critical threshold such as in volumetric muscle loss (VML) resulting from blast injuries, tumour resections, or surgical ablations, the endogenous repair mechanism fails [206]. Instead of regenerating contractile tissue, the defect is replaced by fibrotic scar tissue, resulting in functional impairment, reduced mobility, and aesthetic deformity [207]. Conventional interventions, including autologous muscle flap transfers, are further constrained by donor-site morbidity, ischemic necrosis, and limited vascular integration [208]. In this context, 3D bioprinting offers a powerful approach to engineer biomimetic, cell-laden muscle constructs that reproduce the hierarchical organization, biochemical gradients, and mechanical anisotropy of native skeletal muscle, thereby supporting functional tissue regeneration [209].

A key challenge in engineering functional muscle tissue lies in ensuring adequate oxygenation and metabolic support within clinically relevant construct volumes. To address this, a magnesium peroxide (MgO_2_)-incorporated bio-ink composed of thiolated and maleimide-conjugated gelatin was formulated to support C2C12 myoblasts (1.0×10^6^ cells/ml) through *in situ* crosslinking and sustained release of Mg^2+^ ions and oxygen [210]. Extrusion bioprinting yielded lattice-shaped constructs (20 mm×20 mm×2 mm) with high fidelity and uniform cell distribution. Upon implantation in a murine VML model, these constructs restored muscle mass within seven days and promoted M2 macrophage polarization, although the finite lifetime of MgO_2_ limited prolonged metabolic support in large defects. Building on this concept, a photosynthetically active, electrically stimulated GelMA bio-ink incorporating human adipose-derived stem cells (hASCs, 2.0×10^7^ cells/ml) and the cyanobacterium *Synechococcus elongatus* served as a biological oxygen generator [211]. Electrical stimulation induced uniaxial cell alignment, while light-driven oxygen release sustained metabolic activity. The bioprinted constructs (2.0 mm×4.0 mm×1.5 mm) exhibited 7- to 10-fold upregulation of myogenic genes, enhanced vascularization, and functional regeneration in VML models, though practical limitations such as microbial-mammalian co-cultures and light penetration constrain clinical translation. Together, these studies highlight the importance of metabolic modulation for supporting large muscle constructs.

Beyond oxygen delivery, biochemical recapitulation of the native ECM plays a crucial role in myogenesis. Muscle-derived dECM bio-inks, rich in tissue-specific growth factors and adhesion motifs, have been leveraged to enhance myogenic differentiation. Using a granule-based bioprinting reservoir, a muscle dECM bio-ink containing human skeletal muscle cells (2.0×10^7^ cells/ml) was printed into constructs (15 mm×6 mm×4 mm) that supported de novo myofibre formation and reduced hypoxia [212]. Furthermore, coaxial bioprinting of muscle and vascular dECM bio-inks yielded prevascularized constructs achieving about 85% functional recovery and extensive neurovascular integration in rat VML models [212]. Although dECM variability and mechanical softness necessitate reinforcement with synthetic polymers, these findings highlight how native biochemical cues can direct muscle regeneration. Complementing biochemical mimicry, mechanical programming has emerged as another powerful design parameter. GelMA-fibrinogen IPN bio-inks containing C2C12 myoblasts (2.0×10^5^ cells/ml) printed within a Carbopol support bath produced anisotropic constructs (15 mm×6 mm×4 mm) with tunable stiffness [213]. Optimized constructs demonstrated elongated myotubes, elevated myogenic marker expression, enhanced *in vivo* angiogenesis, and host-cell infiltration highlighting the synergy between mechanical environment and cellular behavior.

**Fig. 5:**
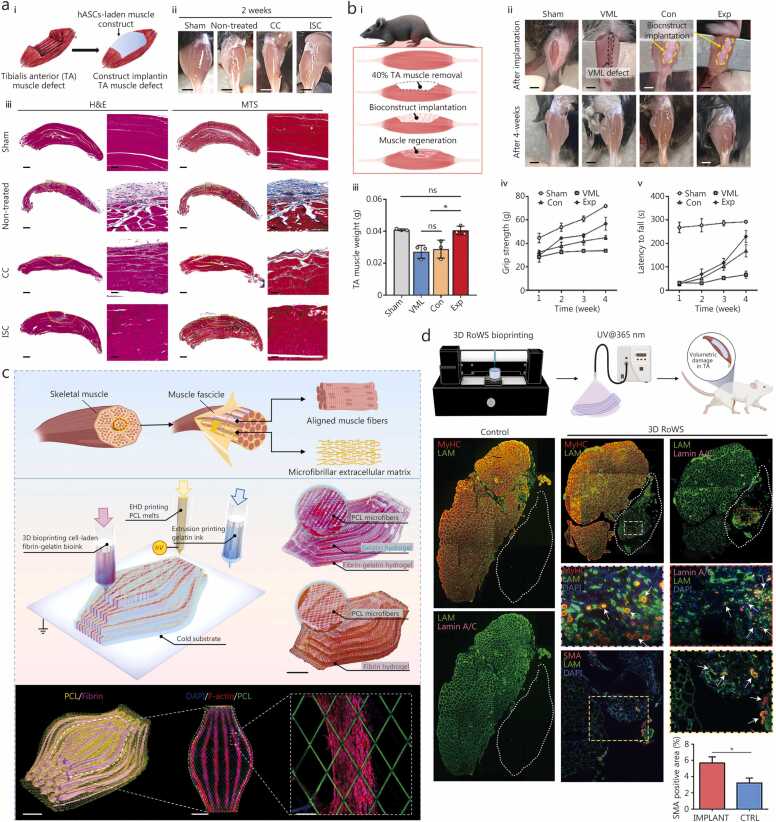
3D bioprinting strategies for VML repair. **a** Aligned cell-laden GelMA structures for VML. (i) Schematic of hASC-laden muscle construct implantation on tibialis anterior (TA) defect. (ii) Optical images of TA (scale bar=2 mm) and (iii) H&E and MTS staining of transplanted sites (yellow dotted line: defect region) (scale bar=50 μm and 500 μm). Reproduced with permission, licensed under CC-BY-4.0 [214]. **b** Muscle functional and histological evaluation in mouse VML model. (i) Schematic of implantation, (ii) gross images of TA immediately and 4 weeks post-implantation (scale bar=2 mm). Quantification of TA muscle weight (iii), grip strength (iv), and latency to fall (v) (*n*=3). Reproduced with permission [216]. **c** Multi-modal bioprinting of microfiber-reinforced living muscle constructs. (Top) Native skeletal muscle anatomy. (Middle) Schematic of microfiber-reinforced muscle constructs via simultaneous printing cell-laden hydrogel, sacrificial hydrogel, and structural PCL microfibers; optical images i) before and ii) after gelatin leaching (scale bar=5 mm). (Bottom) μCT 3D reconstruction and cellular orientation (scale bar=2 mm, 5 mm, and 200 μm). Reproduced with permission [217]. **d** Human PC-derived 3D-biofabricated myo-substitute engraftment in mouse TA VML model. Reproduced with permission, licensed under CC-BY-4.0 [218]. HASCs. Human adipose-derived stem cells; CC. Conventionally crosslinked; CTRL. Control samples; ISC. *in situ* crosslinked; IMPLANT. Implant samples; MTS. Masson’s trichrome staining; H&E. Hematoxylin and eosin; VML. Volumetric muscle loss; Con. Normally bioprinted structure; Exp. Blade-assisted bioprinted structure; EHD. Electrohydrodynamic; PCL. Poly(ε-caprolactone); RoWS. Rotary wet-spinning; DAPI. 4′;6-diamidino-2-phenylindole; PCL. Poly(ε-caprolactone); SMA. Smooth muscle actin; UV. Ultraviolet; TA. Tibialis anterior.

In parallel, researchers have harnessed structural anisotropy to reproduce native muscle fascicular alignment, essential for force generation. *In situ* crosslinking of extrusion-bioprinted GelMA constructs (2.0 mm×4.0 mm×1.5 mm) containing hASCs (1.0×10^7^ cells/ml) under shear flow and instantaneous UV curing induced uniaxial alignment and robust myotube formation (Fig. 5**a**) [214]. Similarly, magnetically assisted bioprinting of iron oxide nanoparticle-laden GelMA constructs (2 mm×4 mm×1 mm) encapsulating hASCs (1.0×10^7^ cells/ml) generated aligned fibres with improved cytoskeletal organization and myogenic gene expression under controlled magnetic fields [215]. These constructs activated Hippo signalling and stretch-activated ion channels, promoting *in vivo* regeneration. A complementary blade-assisted extrusion strategy using collagen-hASC constructs (2.0×10^7^ cells/ml; 2.0 mm×4.0 mm×1.5 mm) further enhanced fibre orientation and restored muscle volume in murine VML defects (Fig. 5**b**) [216]. These alignment-driven strategies demonstrate that biophysical guidance cues are vital for recreating the hierarchical anisotropy characteristic of functional muscle tissue.

As efforts advance toward clinically scalable muscle constructs, maintaining cell viability, alignment, and mechanical integrity across large volumes becomes increasingly critical. Multi-modal fabrication platforms integrating polymeric reinforcement with cell-laden hydrogels have emerged to address these challenges [217]. A multi-modal EHD printing and extrusion printing system co-deposited polymer microfibres, sacrificial gelatin, and fibrin-gelatin bio-inks containing C2C12 cells (2.5×10^6^ cells/ml), yielding composite constructs (outer/inner ∅ 30/15 mm; thickness 6 mm) with tunable stiffness, anisotropic remodelling, and robust myotube formation (Fig. 5**c**) [217]. Extending this concept, the 3D rotary wet-spinning (RoWS) platform continuously produced core-shell hydrogel fibres (about 350 µm diameter) encapsulating human skeletal-muscle-derived PCs (2.0×10⁷ cells/ml) in a PEG-fibrinogen matrix at high output (approximately 4.2 m/min) [218]. The coaxial spinning process generated decimetre-scale aligned bundles that closely mimicked native fascicular organization while minimizing shear injury and enhancing oxygen diffusion. When implanted in mouse VML models, RoWS constructs achieved strong engraftment, neovascularization, and spontaneous contraction, highlighting their translational potential (Fig. 5**d**). The continuous fibre output of RoWS thus represents a clinically scalable pathway toward transplantable muscle grafts, and future integration with multi-omics platforms (proteomics, transcriptomics, metabolomics) could provide mechanistic insights into muscle regeneration.

In summary, 3D bioprinting is converging biochemical, mechanical, architectural, and metabolic strategies to overcome the multifactorial challenges of skeletal muscle regeneration. Advances now integrate vascularization, fibre alignment, mechanotransduction, and oxygen delivery within scalable and mechanically robust constructs. These convergent innovations mark a shift from proof-of-concept models toward clinically viable platforms capable of restoring muscle structure and function following severe VML or other complex musculoskeletal injuries, an outcome of particular significance for military medicine and trauma rehabilitation.

### 5.3 Cartilage injuries

Articular cartilage exhibits inherently limited self-repair capacity due to its avascular, aneural, and alymphatic nature. Consequently, traumatic injury or degenerative diseases often result in fibrocartilaginous tissue with inferior biochemical and mechanical properties compared to native hyaline cartilage [219]. Conventional interventions such as microfracture, autologous chondrocyte implantation, and osteochondral grafting are constrained by donor-site morbidity, poor integration with surrounding cartilage, and progressive degeneration of repair tissue over time [220]. To overcome these challenges, 3D bioprinting has emerged as a promising platform for restoring localized cartilage defects. By spatially organizing chondrocytes or mesenchymal stem cells (MSCs) within biomimetic bio-inks, bioprinting enables the recreation of zonal architecture, biochemical gradients, and mechanical anisotropy [221], [222]. Through precise structural control, these constructs increasingly emulate the organization and functionality of native cartilage.

A scalable strategy integrating melt electrowriting (MEW) with inkjet bioprinting produced stratified, load-bearing scaffolds from PCL micro-fibres (approximately 7 µm fibre diameter, >80% porosity) jetted with porcine MSCs (3.0×10^7^ cells/ml; about 3.5×10^4^ cells per microchamber) [223]. Within 48 h, MSCs self-assembled into approximately 270 µm spheroids that fused into continuous, ECM-rich tissue by day 21, characterized by abundant collagen II deposition. When scaled up to 60 mm×60 mm scaffolds and cultured for 8 weeks in chondrogenic media, the constructs achieved compressive moduli of 180 kPa and 388 kPa at 20% and 30% strain, respectively, and dynamic moduli up to 2.6 MPa, closely approximating native cartilage’s tension-compression response. This hybrid MEW-inkjet approach thus represents a robust and scalable platform for large-area cartilage regeneration.

To further incorporate native ECM bioactivity, a cartilage-derived dECM bio-ink composed of 5% dECM powder, 5% w/v GelMA, and 2% w/v alginate was developed and laden with infrapatellar fat pad-derived ADSCs (1.0×10^7^ cells/ml) [224]. Extrusion-printed cylindrical constructs (∅ 20 mm×4 mm) displayed uniform cell distribution, high viability, and upregulated SRY-box transcription factor 9 (SOX9), collagen, type II, alpha 1 chain (COL2A1), and aggrecan (ACAN) expression. Histological analyses [Safranin O, toluidine blue, and hematoxylin & eosin (H&E)] confirmed glycosaminoglycan- and collagen II-rich ECM formation. When implanted into rabbit femoral trochlear defects (3.5 mm×3.0 mm), the constructs achieved complete defect restoration within 12 weeks, producing smooth, hyaline-like cartilage and superior histological scores compared with cell-only or scaffold-only controls.

An ultrafast, light-based technique termed FLight biofabrication further demonstrated the potential for high-throughput, minimally invasive cartilage repair [89]. Using a photo-crosslinkable hyaluronic acid-norbornene (HA-NB) and bifunctional thiolated PEG bio-ink containing human infant polydactyly chondrocytes (5.0×10^6^ cells/ml), zonally organized tissue constructs (2 mm×4 mm) were printed within 3.3 s, forming highly aligned structures (8 µm filaments and 14 µm micro-channels) that guided ECM orientation. The constructs maintained >85% viability for 8 weeks and reached approximately 85% of native glycosaminoglycan (GAG) content, with stable chondrogenic gene expression (SOX9, COL2A1, and ACAN) and minimal hypertrophic markers [low runt-related transcription factor 2 (RUNX2)]. Over 56 d, compressive modulus increased from about 7 kPa to 1 MPa, and implanted constructs exhibited collagen II alignment comparable to native cartilage, highlighting the potential of light-based bioprinting for rapid, on-demand cartilage restoration.

To replicate the native tissue’s depth-dependent organization, a dual-factor releasing gradient bioprinted construct combined PCL scaffolds with MSC-laden hydrogel (approximately 1.0×10^7^ cells/ml) containing poly(lactic-co-glycolic acid) microspheres loaded with TGF-β3 (20 ng/ml) and bone morphogenic protein (BMP)-4 (100 ng/ml) [225]. Layer-specific pore spacing (150–750 µm) supported ≥95% cell viability and zone-specific differentiation, with proteoglycan 4 (PRG4) and COL2A1 expressed in superficial layers and COL10A1 and RUNX2 in deeper regions. Toluidine blue and Safranin O staining confirmed proteoglycan-rich ECM, while the mechanical modulus approached that of native cartilage. In full-thickness rabbit knee defects (4 mm×4 mm×4 mm), the constructs facilitated hyaline-like tissue regeneration with restored zonal architecture and significantly improved histological outcomes.

These advances illustrate how biomaterial innovation, architectural precision, and biochemical guidance converge in cartilage bioprinting to recapitulate the zonal and load-bearing characteristics of native tissue. By integrating gradient-based cues, ECM bioactivity, and mechanical maturation, modern 3D bioprinting platforms are establishing a clinically translatable framework for high-fidelity cartilage repair, offering transformative potential for both civilian and military medicine.

### 5.4 Bone injuries

Bone possesses substantial intrinsic regenerative potential through coordinated osteoblast activity, vascular invasion, and remodelling. However, this capability fails under extensive trauma or critical-sized defects [226]. Conventional interventions such as autografts, allografts, and inert implants are constrained by donor-site scarcity, immune incompatibility, and poor structural integration [227]. In this context, 3D bioprinting provides a transformative framework for regenerating bone by spatially organizing osteogenic cells, growth factors, and bioactive ceramics within mechanically stable, anatomically tailored scaffolds [228]. Through architectural precision and controlled biological guidance, bioprinting enables fabrication of vascularized, mineralizing constructs that restore both mechanical integrity and biological functionality across craniofacial and long-bone defects.

Guided by developmental principles, one strategy engineered osteo-callus organoids from human bone marrow-derived stem cells (BMSCs) encapsulated within DLP-printed GelMA microspheres to recapitulate endochondral ossification [229]. BMSCs (2.0×10^6^ cells/ml) embedded in 10% w/v GelMA formed microspheres of 200–600 μm diameter, with 400 μm yielding optimal viability and cell density. After 21 d of chondrogenic induction, microspheres self-assembled into organoids exhibiting sequential proliferation, chondrogenic maturation, hypertrophy, and osteogenic differentiation, as evidenced by SOX9, collagen type II (COL2), RUNX2, and OSX expression, vascular endothelial growth factor (VEGF) upregulation, and bulk RNA-sequencing. Structural maturation confirmed by micro-CT, histology, and transmission electron microscopy (TEM) revealed organized collagen fibrils and mineral deposition. When approximately 3000 organoids were implanted into rabbit femoral defects (5 mm×4 mm), rapid vascular infiltration and trabecular bone formation achieved near-complete defect closure within 4 weeks, significantly outperforming undifferentiated or scaffold-only controls. These findings highlight organoid-based developmental programming as an efficient route for orchestrating endochondral ossification and rapid defect healing.

For craniofacial bone repair, a recent approach enabled on-demand fabrication of autologous bone (AB) particle-reinforced scaffolds integrated with BMSCs [230]. Using extrusion-based printing, a dual-ink system combined 5% alginate, 5% gelatin, 25% w/v AB particle (Alg-Gel-AB) composite biomaterial-ink with a BMSC-laden Alg-Gel bio-ink (2.5×10^6^ cells/ml) to produce 20.0 mm×20.0 mm×2.1 mm constructs crosslinked in 3% CaCl_2_ solution. A PCL shell mimicked cortical bone, providing enhanced mechanical stability. The constructs maintained >90% viability over 21 d, exhibited elevated alkaline phosphatase (ALP) activity and osteogenic gene expression [*RUNX2*, *BMP2*, osteocalcin (*OCN*)], and showed organized ECM via SEM. In 2-cm beagle cranial defects, micro-CT and histology (H&E, Masson’s trichrome, Safranin O-Fast Green) revealed extensive osteoid and mature bone formation after 9 months. Immunohistochemistry confirmed COL1, OCN, ACAN, and COL2 expression, while GFP-labelled BMSC differentiated into osteogenic, chondrogenic, and vascular lineages. Enhanced stromal cell-derived factor 1 (SDF1)-mediated BMSC recruitment and immunomodulation [ ↑interleukin-10 (IL-10), ↓TNF-α] further promoted regeneration, validating the use of AB particle-reinforced scaffolds as a clinically adaptable and immuno-tolerant platform for autologous bone repair.

Further advancing the bioactive microenvironments, prevascularized bone organoids were fabricated by co-culturing MSCs and HUVECs (6.0×10^5^ cells/ml each) with graphene oxide (GO) microparticles (1.2×10^6^ per microwell) to form multicellular aggregates (116–133 μm in diameter) [231]. GO incorporation improved cell adhesion, reduced agglomeration, and promoted sustained proliferation, as confirmed by live/dead staining and CCK-8 assays. These GO-laden organoids demonstrated significantly elevated expression of osteogenic markers [(ALP, COL1, BMP2, RUNX2, and osteopontin (OPN)], robust mineralization by Alizarin Red staining, and pronounced CD31 expression confirming self-organized vascular formation. When encapsulated in 7.5 wt% GelMA hydrogel and bioprinted to match rat cranial defects (4 mm×1 mm), the prevascularized bone organoids (M+H+GO) achieved superior *in vivo* outcomes, with bone volume fraction (BV/TV) reaching (32.28±4.48)% (2.1–4.1 times higher than control groups) and the highest bone mineral density at 8 weeks post-implantation. Enhanced OCN (6.11±0.27)% and CD31 (3.77±0.21)% expression confirmed rapid osteogenesis and angiogenesis, while immunofluorescence analysis showed a favourable M2 macrophage polarization ratio (2.06±0.03), indicating attenuated inflammation. The GelMA-encapsulated, GO-mediated prevascularized bone organoids enabled rapid, vascularized, and immunomodulated bone regeneration, offering a promising cell-instructive platform for critical-sized cranial defect repair in translational and military medicine contexts.

To address the persistent challenge of vascularization in large defects, vascularized bone constructs were fabricated by alternating deposition of 5 wt% GelMA bio-ink loaded with BMSCs (5.0×10^6^ cells/ml) and 10 wt% PLA-PEG-PLA sacrificial bio-ink loaded with rat aortic ECs (5.0×10^6^ cells/ml) using an extrusion-based bioprinting system [232]. The approach produced 10.0 mm×10.0 mm×2.5 mm constructs with interconnected tubular channels. Upon photo-crosslinking, the sacrificial PLA-PEG-PLA copolymer ink dissolved at 37 °C, yielding uniform endothelial coverage with perfusable channels. This configuration enhanced angiogenesis gene expression [*CD31*, V*EGF*, platelet-derived growth factor (*PDGF)*, hypoxia-inducible factor-1α (*HIF-1α*)], facilitated endothelial migration and vessel formation, and synergistically promoted osteogenesis with increased ALP, Ca^2+^ deposition, and RUNX2, OPN, Osterix, and Col1A1 expression. Transcriptomic profiling revealed coupling of angiogenic and osteogenic pathways. Implantation into 5-mm calvarial defects achieved complete bridging with high vascular density, as confirmed by micro-CT, histology, and CD31/vWF staining, highlighting the importance of vascular induction for robust bone regeneration.

These bioprinting strategies converge on a unified goal, engineering mechanically competent, biologically active, and vascularized bone constructs capable of regenerating complex skeletal defects. By integrating developmental organoid programming, autologous cell-particle composites, prevascularized microtissues, and vascular guidance, 3D bioprinting is redefining the therapeutic landscape for bone repair, advancing toward clinically translatable, large-scale reconstruction in both civilian and military medicine.

## 6 Challenges and future directions

3D bioprinting has rapidly progressed from proof-of-concept demonstrations to a versatile platform for regenerative medicine. As discussed in earlier sections, advances in bio-ink design and bioprinting techniques have established a strong technological foundation. However, the next phase of innovation should go beyond incremental improvements in materials and hardware. Future progress will depend on integrating complementary fabrication modalities, harnessing computational intelligence, and overcoming persistent biological and translational barriers to achieve clinically relevant outcomes.

A primary challenge lies in replicating the intricate structural and cellular complexity of native tissues, which are characterized by heterocellular composition, biochemical gradients, and hierarchical organization [8]. Achieving this level of complexity remains beyond the capabilities of any single bioprinting modality. To bridge this gap, multi-modal bioprinting systems have emerged as a promising solution. By combining complementary techniques within unified platforms, these systems can fabricate complex, multi-material constructs with enhanced functionality [233], [234]. They can accommodate a wide range of material viscosities and cell types, facilitating the fabrication of tissue constructs that more closely mimic native biology. Commercial platforms from Cellink, Allevi, Regemat, and RegenHU exemplify this approach by enabling independent multi-printhead configurations for simultaneous deposition of diverse materials and cell types in a single construct [235]. These capabilities can be further augmented by integrating supplementary techniques such as electrospinning and melt electrowriting, which introduce submicron fibers essential for cell alignment, ECM reinforcement, and multi-scale structural features [123], [223], [236], [237], [238]. Notable demonstrations include multi-modal inkjet/electrospinning systems that generate cartilage-like constructs with superior mechanical performance and *in vivo* maturation [239], inkjet-melt extrusion approaches producing large, stratified articular cartilage constructs with native-like collagen organization and a 50-fold increase in compressive modulus [223], and LIFT-extrusion-based printing yielding dermo-epidermal skin constructs with high *in vivo* engraftment rates [240]. Furthermore, the integration of two-photon ablation with volumetric bioprinting has enabled the fabrication of perfusable microcapillary networks at sub-cellular resolution, highlighting the transformative potential of multi-modal strategies in achieving physiologically relevant complexity [241]. Moving forward, efforts must focus on enhancing these systems through seamless integration of multi-material, multi-cellular, and multi-scale features, coupled with robust, reproducible, and scalable printing strategies.

Complementing these hardware advancements, the inherent complexity of bioprinting processes introduces additional critical challenges. Each process involves numerous interdependent parameters, including bio-ink rheology, nozzle mechanics, printing settings, and post-fabrication cellular responses that collectively determine print fidelity, functionality, and reproducibility [242]. Traditional modelling approaches often struggle to capture the non-linear relationships within such high-dimensional data. ML offers a powerful alternative, capable of deciphering complex relationships and enabling optimization of bioprinting workflows [243], [244], [245].

ML, a subset of artificial intelligence, enables systems to learn from data, recognize patterns, and make intelligent decisions[246], [247]. There are 4 types of ML algorithms: supervised learning [248], unsupervised learning [249], [250], semi-supervised learning [251], and reinforcement learning [252], [253], [254]. Supervised learning relies on labelled datasets (input-output pairs) to map inputs to outputs, allowing predictions on new data. Although it requires considerable effort and domain knowledge to label data and define objectives, supervised learning can achieve high predictive accuracy with sufficient training [248]. Unsupervised learning, in contrast, works with unlabeled datasets to uncover patterns or relationships within complex data without human guidance [249], [250]. Semi-supervised learning combines aspects of both approaches, training on datasets containing both labeled and unlabeled data, thereby improving model performance while reducing reliance on costly labeled datasets [251]. Finally, reinforcement learning employs a trial-and-error approach, wherein the algorithm iteratively explores actions, parameters, and outcomes to identify strategies that maximize performance [252], [253], [254].

ML has already shown promise in various bioprinting applications, ranging from parameter optimization in extrusion-based [255], inkjet-based [256], and vat photopolymerization-based systems [257], to predictive modelling of cell viability and proliferation [258], [259]. For example, ML models have accurately predicted cell viability based on printing pressure, nozzle size, and crosslinking conditions [259], and have estimated cell numbers in inkjet bioprinting by analyzing droplet velocity profiles [260]. Such approaches accelerate iterative design cycles and pave the way for closed-loop, self-optimizing bioprinting systems driven by real-time feedback. Nevertheless, significant hurdles remain, including the scarcity of large, standardized training datasets and the need for interpretable, regulatory-compliant ML models to facilitate clinical translation.

Ultimately, the long-term success of 3D bioprinting will hinge on overcoming biological and translational barriers beyond the realm of fabrication. A critical aspect involves the selection of appropriate cell sources. Stem cell technologies such as iPSCs offer a virtually infinite reservoir of cells capable of differentiating into multiple lineages [261]. However, their clinical translation remains challenging due to limited differentiation efficiency, high culture costs, and the persistence of residual undifferentiated cells [262]. These undifferentiated populations raise significant safety concerns, as their uncontrolled proliferation may lead to tumorigenicity or other unintended cellular behaviours within engineered constructs. Addressing these risks through improved differentiation protocols, purification strategies, and long-term safety assessments will be essential for the safe clinical implementation of iPSCs-derived tissues. In addition to cell source optimization, other key barriers include establishing functional vascular networks to sustain thick, metabolically active tissues; developing immune-compatible bio-inks to prevent host rejection; and ensuring compliance with stringent regulatory frameworks.

To date, several 3D bioprinted tissues have reached early stages of clinical translation, with a notable milestone being the transplantation of the first autologous bioprinted ear implant in 2022 [263]. While this case highlights the transformative potential of bioprinting to create patient-specific, functional tissue constructs, it has not yet received full regulatory approval, illustrating the significant barriers that remain for clinical translation. These barriers include regulatory approval, standardization, and manufacturing readiness. Robust, reproducible datasets demonstrating sterility, safety, and functionality are essential, and there are several standards guides from International Organization for Standardization (ISO) and American Society for Testing and Materials (ASTM) in this regard. For instance, ASTM F3659-24 offers a framework for evaluating bio-ink composition, sterility, and performance [264], while ASTM efforts such as WK83109 [265] and WK78224 [266] provide guidance for design and mechanical characterization of vat photopolymerization-fabricated constructs. More broadly, standards such as ISO 10993-1:2025 establish principles for biological safety testing [267], and ISO 22442-1:2020 supports Good Manufacturing Practice (GMP)-compliant manufacturing and quality management [268]. Integration of GMP is critical, encompassing raw material qualification, batch traceability, validated aseptic handling, and process control. An example is X-Pure® GelMA, the first GMP-produced GelMA bio-ink, which offers low endotoxin levels and full traceability [269]. Currently, only a limited number of facilities are equipped to fabricate viable tissue constructs under GMP conditions, often requiring careful transport of patient-derived cells while maintaining high viability. Overall, while early clinical demonstrations are encouraging, broader adoption of bioprinting will require harmonization of standards, availability of GMP-grade bio-inks, robust process controls, and long-term validation of safety and functionality. Aligning technological innovation with regulatory clarity and standardized manufacturing practices will be essential for bioprinting to transition from promising laboratory research to reproducible, scalable, and clinically relevant therapies.

In summary, the future of 3D bioprinting lies in the seamless integration of advanced engineering, biological insight, and regulatory compliance. Multi-modal bioprinting and ML-driven optimization will enable precise spatial control of cells and biomaterials, advancing the fabrication of physiologically relevant tissue constructs. At the same time, overcoming biological barriers such as efficient stem cell differentiation, vascularization, and immune compatibility remains essential for functional and safe tissue constructs. Establishing standardized, GMP-compliant manufacturing pipelines and clear regulatory frameworks will further enhance reproducibility and clinical readiness. Collectively, these developments will accelerate the translation of 3D bioprinting from experimental research to clinically relevant, patient-specific regenerative therapies.

## 7 Conclusions

3D bioprinting has rapidly evolved from an emerging concept to a powerful platform capable of fabricating complex, multi-cellular tissue constructs with exceptional spatial precision. Advances in bio-ink development and diverse bioprinting modalities including extrusion-based, inkjet-based, and vat photopolymerization techniques have enabled the creation of functional tissue analogues that capture essential features of native architecture and cell-matrix interactions. These breakthroughs have already expanded the horizons of cardiovascular repair, endocrine and metabolic disease modelling, and neurodegenerative disease research, highlighting the immense potential of this technology.

Nevertheless, substantial challenges remain. Existing bio-inks still balance trade-offs between mechanical robustness, bioactivity, and cytocompatibility, while current platforms struggle to replicate the full complexity of native tissues, including hierarchical organization, vascularization, and dynamic multi-cellular interactions. Furthermore, scalability, long-term functionality, and the absence of standardized protocols continue to hinder reproducibility and clinical translation.

Looking ahead, future progress will depend on uniting multi-modal bioprinting strategies with next-generation bio-inks that are both mechanically tunable and biologically functional, supported by computational and machine learning tools to optimize processes and guide post-printing maturation. Equally critical is addressing translational barriers such as immune compatibility, stable vascularization, and regulatory compliance to ensure that engineered tissues can safely and effectively reach patients.

By overcoming these challenges, 3D bioprinting holds the promise of revolutionizing regenerative medicine. It can provide physiologically relevant disease models that accelerate therapeutic discovery, deliver life-saving implants tailored to individual patients, and ultimately enable the on-demand fabrication of fully functional human tissues and organs. Such progress would not only redefine how we treat disease but also transform the very future of healthcare shifting the paradigm from repair to true regeneration.

## Abbreviations

2D: Two-dimensional

3D: Three-dimensional

BMSCs: Bone marrow-derived stem cells

CaCl_2_: Calcium chloride

dECM: Decellularized extracellular matrix

DLP: Digital Light Processing

DNA: Deoxyribonucleic acid

E: Electric field strength

ECs: Endothelial cells

ECM: Extracellular matrix

EHDJ: Electrohydrodynamic jetting

FBs: Fibroblasts

FLight: Filamented light

GelMA: Gelatin methacryloyl

GO: Graphene oxide

GSIS: Glucose-stimulated insulin secretion

HAMA: Hyaluronic acid methacrylate

HICA: Human islet-like cellular aggregate

hiPSCs: Human induced pluripotent stem cells

HUVECs: Human umbilical vein endothelial cells

KCs: Keratinocytes

KSM: Kenics static mixer

LAP: Lithium phenyl-2,4,6-trimethylbenzoylphosphinate

LIFT: Laser-induced forward transfer

MSCs: Mesenchymal stem cells

PAs: Photo-absorbers

PCs: Pericytes

PEG: Poly(ethylene glycol)

PEGDA: Poly(ethylene glycol)-diacrylate

PIs: Photo-initiators

PVA: Polyvinyl alcohol

Q: Flow rate

ROS: Reactive oxygen species

RoWS: Rotary wet-spinning

UV: Ultraviolet

VML: Volumetric muscle loss

## Ethics approval and consent to participate

Not applicable.

## Funding

Not applicable.

## Data Availability

Not applicable.
